# Metabolic characterization of colorectal cancer cells harbouring different *KRAS* mutations in codon 12, 13, 61 and 146 using human SW48 isogenic cell lines

**DOI:** 10.1007/s11306-020-01674-2

**Published:** 2020-04-16

**Authors:** Dorna Varshavi, Dorsa Varshavi, Nicola McCarthy, Kirill Veselkov, Hector C. Keun, Jeremy R. Everett

**Affiliations:** 1 Medway Metabonomics Research Group, University of Greenwich, Chatham Maritime, Kent, ME4 4TB UK; 2 Horizon Discovery Ltd., Cambridge Research Park, 8100 Beach Dr, Waterbeach, Cambridge, CB25 9TL UK; 3 Department of Surgery and Cancer, Faculty of Medicine, Imperial College, London, SW7 2AZ UK; 4 Department of Surgery and Cancer, Imperial College London, Hammersmith Hospital Campus, London, W12 ONN UK

**Keywords:** KRAS, Mutations, Colorectal cancer, Metabonomics, Metabolomics, Metabolic profiling, NMR

## Abstract

**Introduction:**

Kirsten Rat Sarcoma Viral Oncogene Homolog (*KRAS*) mutations occur in approximately one-third of colorectal (CRC) tumours and have been associated with poor prognosis and resistance to some therapeutics. In addition to the well-documented pro-tumorigenic role of mutant Ras alleles, there is some evidence suggesting that not all *KRAS* mutations are equal and the position and type of amino acid substitutions regulate biochemical activity and transforming capacity of *KRAS* mutations.

**Objectives:**

To investigate the metabolic signatures associated with different *KRAS* mutations in codons 12, 13, 61 and 146 and to determine what metabolic pathways are affected by different *KRAS* mutations.

**Methods:**

We applied an NMR-based metabonomics approach to compare the metabolic profiles of the intracellular extracts and the extracellular media from isogenic human SW48 CRC cell lines with different *KRAS* mutations in codons 12 (G12D, G12A, G12C, G12S, G12R, G12V), 13 (G13D), 61 (*Q61H*) and 146 (*A146T*) with their wild-type counterpart. We used false discovery rate (FDR)-corrected analysis of variance (ANOVA) to determine metabolites that were statistically significantly different in concentration between the different mutants.

**Results:**

CRC cells carrying distinct *KRAS* mutations exhibited differential metabolic remodelling, including differences in glycolysis, glutamine utilization and in amino acid, nucleotide and hexosamine metabolism.

**Conclusions:**

Metabolic differences among different *KRAS* mutations might play a role in their different responses to anticancer treatments and hence could be exploited as novel metabolic vulnerabilities to develop more effective therapies against oncogenic *KRAS.*

**Electronic supplementary material:**

The online version of this article (10.1007/s11306-020-01674-2) contains supplementary material, which is available to authorized users.

## Introduction

*KRAS* is a gene that has the potential to cause cancer when it is mutated i.e. it is an oncogene. Point mutations in the *KRAS* gene are present in approximately 35–45% of colorectal cancers (Dinu et al. 2014; Tan and Du 2012), and serve as a negative predictive factor of response to anti-EGFR therapy (Lievre et al. 2006). These mutations lead to the loss of intrinsic GTPase activity and therefore to increased proliferation and resistance to apoptosis (Jia et al. 2017). The majority of *KRAS* mutations occur in codons 12 and 13, while other mutations, such as those in codons 61 and 146 are less common (Guarnaccia et al. 2018; Margonis et al. 2015; Stolze et al. 2015). The most common point mutations in codon 12 and 13 are codon 12 Gly → Asp (G12D), codon 12 Gly → Val (G12V), and codon 13 Gly → Asp (G13D) substitutions (Seekhuntod et al. 2016). It is well known that not all *KRAS* mutations are equivalent and the position and type of amino acid substitutions regulate both the biochemical activity and transforming capacity of *KRAS* mutations (Brunelli et al. 2014). For example, it has been reported that mutations of *KRAS* at codon 12 have greater transforming ability than at codon 13 and are linked with a more aggressive cancer phenotype, although, patients with codon 13 mutations exhibit a markedly worse prognosis (Yokota 2012). Moreover, it has been suggested that different *KRAS* mutations display different responses to treatments. For instance, patients with G13D mutations have been shown to respond better to anti-EGFR therapy compared to patients with other *KRAS* mutations (Kishiki et al. 2014; Stolze et al. 2015; Tural et al. 2013).

Oncogenic *KRAS* has been shown to promote the decoupling of glycolysis and TCA metabolism, with glutamine fuelling the TCA cycle as an alternative source of carbon (Son et al. 2013). These findings support the notion that oncogenic *KRAS* plays a key role in the metabolic reprogramming of cancer cells (Vizan et al. 2005; Ying et al. 2012). Moran et al. found that *KRAS* mutation was associated with increased dependency on folate metabolism in non-small cell lung cancer cells (Moran et al. 2014). A panel of *KRAS* mutants in isogenic SW48 colon cancer cell lines exhibited a unique signalling network signature and proteome expression profile (Hammond et al. 2015). In addition, Kerr et al. demonstrated that homozygous *KRAS*^G12D/G12D^ cells had a glycolytic switch and increased channelling of glucose-derived metabolites into the TCA cycle and glutathione biosynthesis (Kerr et al. 2016). However, in spite of all this work, the overall metabolic dysregulation driven by different *KRAS* mutations in colorectal cancer is still not clear.

Since metabolic reprogramming is a hallmark of cancer and differential metabolic reprogramming is important in the development and selection of effective therapies, we applied an exploratory, NMR-based metabonomics approach to characterize the metabolic pathways affected in isogenic SW48 cells harbouring different *KRAS* mutations at codons 12, 13, 61 and 146. Not only will this work help explain why different *KRAS* mutations give rise to different cancer metabolism and phenotypic outcomes but also, the identification of metabolic pathways associated with specific *KRAS* mutations may be of use in the design of more effective targeted therapies for colorectal cancer in the future.

## Materials and methods

### Cell culture

Isogenic SW48 colorectal carcinoma cell lines were sourced from Horizon Discovery Ltd (Cambridge, UK). All of these cell lines were generated using a recombinant Adeno-Associated Virus (rAAV)-based gene editing platform according to published methods (Arena et al. 2007; Vartanian et al. 2013). All cell lines were cultured in McCoy’s 5A medium (Gibco, Life Technologies, UK), supplemented with 10% foetal bovine serum (FBS) (Gibco, UK). Cells were grown in 150 cm^2^ cell culture flasks (Corning, UK), incubated at 37 °C in a humidified atmosphere containing 5% CO_2_ and passaged routinely until they reached approximately 80–90% confluence. All experiments were performed in five biological replicates.

### Cell harvesting method

The medium was removed from the attached cells in a T150 cm^2^ flask and the cells were washed once with 20 mL phosphate buffered saline (PBS, pH 7.0–7.3, CaCl_2_- and MgCl_2_-free, Gibco, Life Technologies, UK) at room temperature to remove residual serum. 3 ml 0.05% Trypsin–EDTA (Gibco, Life Technologies, UK) was added to the flasks and these were incubated at 37 °C for 3 min. Once the cells had detached (checked using a phase contrast inverted microscope) 10 mL of complete medium was added to dilute the trypsin. The media containing the detached cells was transferred into a 50 mL conical tube (Fisher Scientific, UK). Cells collected from different flasks were mixed into one tube and then 300 µL of cell suspension was removed for accurate cell counting using a Nucleocounter (Chemometec, Denmark). The cell suspension was centrifuged at 1400 rpm for 5 min at room temperature. After removing the media, the cell pellet was suspended in Opti-MEM media (Gibco, Life Technologies, UK) and aliquots (1 mL) containing 2 × 10^7^ SW48 cells were added in 5 T75 cm^2^ low adherence flasks (Corning, UK) containing 9 mL of Opti-MEM medium. After incubation at 37 °C for 6 h, the cells from each flask were harvested and transferred into a 15 mL Falcon tube (Fisher Scientific, UK). The cell suspension was centrifuged at 1400 rpm for 5 min at 4 °C, the Opti-MEM media was removed and the cell pellet was washed once with 10 mL ice cold PBS to remove extracellular metabolites. After removing PBS by centrifugation (1400 rpm and 4 °C for 5 min), the cell pellets were immediately frozen in liquid nitrogen and stored at − 80 °C. The Opti-MEM media removed from SW48 cells was also snap frozen in liquid nitrogen and stored at − 80 °C.

### Metabolite extraction of SW48 cell lines

Cells were extracted in 1000 μL chilled MeOH/H_2_O (− 20 °C, 80/20 (v/v)) for 30 s at 15 Hz using 2 mL CK28-R reinforced ceramic homogenising tubes and a TissueLyser II (QIAGEN, UK). The samples were centrifuged at 4 °C and 7000×*g* for 5 min and the supernatant was collected into a new Eppendorf tube. The pellet was re-extracted once with 1000 μL cold methanol/water (− 20 °C, 80/20 (v/v)) to produce 2 mL of extract. The supernatants were combined, dried under a gentle flow of N_2_ gas (Labconco RapidVap evaporator) and stored at -80° until NMR analysis.

### Sample preparation for NMR analysis

Cell extracts were resuspended by vortexing in 180 μL phosphate buffer (81/19 (v/v) mixture of 0.1 M K_2_HPO_4_ and NaH_2_PO_4_ in 100% ^2^H_2_O with 0.1 mM 3-(trimethylsilyl)propionic-2,2,3,3-d4 acid sodium salt (TSP), pH 7.4). The buffered samples were then centrifuged at 10,000 rpm for 1 min at 4 °C to remove any suspended particles. After centrifugation, the supernatant was transferred into 3 mm diameter NMR tubes (SampleJet Tube 3.0 × 103.5 mm, Bruker Spectrospin; Z112272) using an electronic syringe (SGE-Analytical Science, UK). In addition, four SW48 *KRAS*^G13D/+^ extracts were pooled for analysis in 5 mm o.d. NMR tubes (NORELL, 508-UP-7), in order to provide greater sensitivity for two-dimensional NMR experiments for metabolite identification.

### Cell culture medium sample preparation for NMR analysis

500 μL of Opti-MEM cell medium collected from SW48 cell lines was mixed with 250 ul of phosphate buffer (81:19 (v/v) mixture of 0.6 M K_2_HPO4 and NaH_2_PO_4_ in 100% ^2^H_2_O, pH 7.4) containing TSP (0.5 mM, Sigma-Aldrich) and sodium azide (9 mM, Sigma-Aldrich). 600 μL samples were then transferred into 5 mm diameter NMR tubes (Norell, S-1.7-500-1) and ^1^H NMR spectra of cell culture media acquired.

### ^1^H NMR spectroscopy

^1^H NMR spectra of the metabolites were acquired on a 600 MHz Bruker Avance spectrometer (Bruker BioSpin GmbH, Rheinstetten, Germany) operating at a temperature of 300.0 K. Standard 1D ^1^H NMR spectra were acquired with a 1D NOESY pulse sequence (RD-90°-*t*1-90°-*t*m-90°-acquire, pulse sequence noesygppr1d) with water suppression applied during the relaxation delay (RD) of 4 s and mixing time (tm) of 10 ms. 128 transients for SW48 culture medium and 256 transients for SW48 cell extracts were collected into 65,536 data points with a spectral width of 20 ppm.

### Two-dimensional NMR analysis of cell extracts

Two-dimensional NMR experiments were performed on representative samples to ensure the unambiguous assignment of the identities of the metabolites. The detailed parameters for the acquisition of the 2D NMR spectra of SW48 *KRAS*^G13D/+^and media of SW48 *KRAS*^+/+^are provided in Supplementary Tables 1 and 2.

### NMR data treatment

Prior to Fourier transformation, each free induction decay (FID) was multiplied by an exponential function equivalent to a 0.3 Hz line broadening, in order to improve the signal-to-noise ratio. The resulting spectra were phase corrected, baseline corrected and referenced relative to the chemical shift of the methyl protons of TSP (0.0 ppm) using TOPSPIN 3.2 (Bruker Biospin, UK). The NMR spectra were imported into Matlab (R2010 b, Mathworks) and analysed using in-house Matlab routines written by the team of Dr K. Veselkov. The regions of the spectra upfield of 0.8 ppm, downfield of 10 ppm and the water region (δ 4.7–5.2) were excluded to eliminate the effects of variable water saturation and background noise. All NMR spectra were normalised according to the “Probabilistic Quotient Normalization (Dieterle et al. 2006)” method in order to compensate for variations in the *overall* concentrations of the samples. In order to correct chemical shift variations between the different samples, due to minor changes in pH, ionic strength and solute composition, NMR spectra were aligned using recursive segment-wise peak alignment (RSPA) method. After alignment, all NMR data were log-transformed to convert multiplicative noise into additive noise (Veselkov et al. 2011).

### Pattern recognition and multivariate data analysis

Pattern recognition analyses were performed as described previously (Varshavi et al. 2018). Initially, NMR spectral data were subjected to principal component analysis (PCA) to visualize the general structure of each data set and to identify sub-groups and any potential outliers within the data. Subsequently, a supervised multivariate analysis known as maximum margin criterion (MMC) was applied to simultaneously maximize the inter-group differences i.e. cell genotypes, whilst minimising within group differences i.e. biological replicates (Veselkov et al. 2014). The validity of the MMC models was checked by performing a “leave-one-out” cross-validation using the quadratic as a classifier. One-way analysis of variance (ANOVA) with a false discovery rate (FDR) of 0.1 i.e. 10% to account for multiple hypothesis testing (Benjamini 2010) was employed to determine metabolites that individually (irrespective of other metabolites) have a different abundance between groups. The FDR approach was used in order to avoid the well-known problems that can arise due to multiple hypothesis testing in high dimensional datasets (Benjamini 2010; Han et al. 2009).

### Metabolite identification

Statistically significantly discriminating metabolites were identified by (i) comparison with reference spectra from the Human Metabolome Database (HMDB https://www.hmdb.ca/) (Wishart et al. 2007) and the Biological Magnetic Resonance Data Bank (BMRB, https://www.bmrb.wisc.edu/metabolomics/); (ii) analysis of previously published data; and (iii) the interpretation of a series of two-dimensional (2D) spectra such as 2D ^1^H COSY, J-resolved, HSQC and HMBC, using published methods(Dona et al. 2016; Everett 2015).

## Results

### Intracellular metabolic profiling of isogenic, human colorectal cancer cells harbouring different *KRAS* mutations in codon 12 and 13

**Fig. 1:**
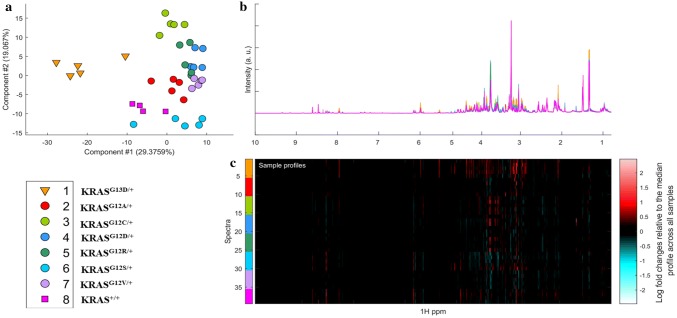
**a** PCA scores plot of the 600 MHz ^1^H NMR spectra from extracts of SW48 cells with *KRAS* mutations in codons 12 and 13 and their wild type counterpart, KRAS^+/+^; **b** superimposed NMR spectra of SW48 cells, with the same colour coding as in the PCA plot, part (**a**); **c** ‘the corresponding heat map display of the 600 MHz ^1^H NMR spectra of SW48 cell lines from 0.8 to 10.0 ppm. Red and blue elements in the spectra indicate NMR signals that are more intense, or less intense, respectively, than the median signal intensity for all the samples

An unsupervised PCA scores plot of all biological replicates of all SW48 cell lines expressing either wild-type or mutant KRAS in either codon 12 or 13 (Fig. 1) showed good clustering and separation of each mutant from *KRAS* WT, with the most variability observed between G13D and the codon 12 *KRAS* mutants and the wild-type. These data indicate that each activating *KRAS* mutation generates a unique metabolic signature that is distinct from KRAS WT. Representative ^1^H NMR spectra of three of these isogenic cell lines are shown in Supplementary Fig. 1.

The supervised dimension reduction technique, maximum margin criterion (MMC) (Veselkov et al. 2014) showed somewhat improved class separation (Supplementary Fig. 2). Correct classification rates of 100% were observed for all models. Leave-one-out cross-validation with the quadratic as a classifier was applied (Supplementary Fig. 3).

**Fig. 2:**
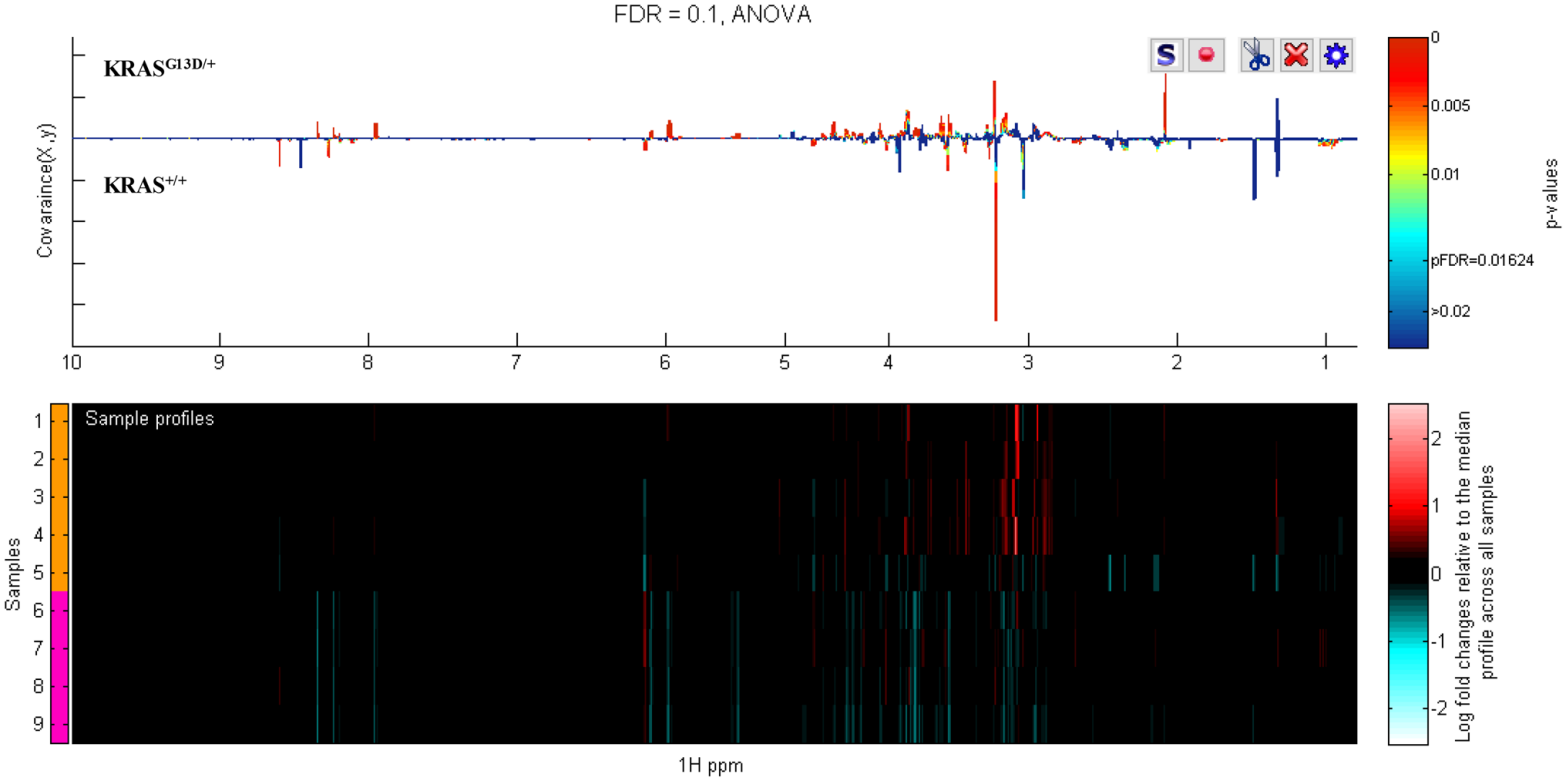
At bottom a ‘heat map display of the 600 MHz ^1^H NMR spectra of *KRAS*^G13D/+^ (top 5 strips) vs the corresponding spectra of parental cell line (*KRAS*^+/+^) (bottom 4 strips). Red and blue elements in the spectra indicate NMR signals that are more intense, or less intense, respectively, than the median signal intensity for all the samples. At top, the corresponding ANOVA plot, showing positive peaks for those metabolite signals that are more intense in *KRAS*^G13D/+^, and negative peaks for those metabolite signals that are less intense. The signals are colour coded by the p value adjusted for an FDR of 0.1. The FDR-adjusted p value for statistically significant difference, pFDR, is 0.016 in this case

One-way ANOVA with a false discovery rate (FDR) of 10%, was used to determine the statistically significantly different metabolites between the *KRAS* mutant cells and their KRAS wild type isogenic cell lines (Fig. 2 and Supplementary Figs. 4–9). Table 1 lists the statistically significant discriminating metabolites and their associated HMDB identification numbers. G12V displayed the most marked metabolic remodelling. All metabolites are identified at MSI Level 2 (Sumner et al. 2007) and confidently identified by MICE methodology (Everett 2015). Comparison of relative fold-changes in the significantly altered metabolites in each KRAS mutant with their WT counterpart showed that KRAS mutations mostly reduced the amount of metabolites relative to WT (Table 2 and Supplementary Tables 3–8).

**Table 1:** Summary of the most significant metabolites differentiating between SW48 cells with *KRAS* mutations in codons 12 and 13 and their wild type counterpart

Metabolite	^1^H NMR parameters: chemical shift in ppm (multiplicity, d doublet, m multiplet, s singlet, t triplet)	*G13D/*+ vs +*/*+	*G12S/*+ vs +*/*+	*G12A/*+ vs +*/*+	*G12C/*+ vs +*/*+	*G12D/*+ vs +*/*+	*G12V/*+ vs +*/*+	*G12R/*+ vs +*/*+
Isoleucine HMDB000172	0.943 (t), 1.01 (d), 3.675 (d)	↓	–	↓	↓	↓	↓	–
Leucine HMDB00687	0.961 (d), 0.972 (d), 1.691 (m), 1.720 (m), 1.748 (m), 3.737 (dd)	↓	–	↓	↓	↓	↓	–
Valine HMDB0000883	0.996 (d), 1.046 (d), 3.615 (d)	↓	–	↓	↓	↓	↓	–
Lactate HMDB00190	1.331 (d), 4.113 (q)	–	–	–	–	↓	↓	–
Threonine HMDB0000167	1.335 (d), 3.591 (d)	–		–	↓	↓	↓	–
Alanine HMDB0000161	1.485 (d), 3.787 (q)	–	–	–	↓	–	↓	–
UDP-*N*-acetyl-glucosamine HMDB00290	2.084 (s), 4.295 (m), 4.367 (m), 5.523 (dd), 5.975 (d), 5.991 (d), 7.958 (d)	↑	↓	↓	–	↓	↓	↓
UDP-*N*-acetyl-galactosamine HMDB0000304	2.088 (s), 3.770 (m), 4.255 (m), 5.556 (dd), 5.975 (d), 5.990 (d)	↑	↓	↓	–	↓	↓	↓
Glutamate HMDB0000148	2.059 (m), 2.140 (m), 2.355 (m), 3.761 (dd)	↓	↓	–	↓	–	↓	–
Glutamine HMDB0000641	2.145 (m), 2.460 (m), 3.783 (t)	–	↓	↓	↓	↓	↓	↓
Glutathione HMDB0000125	2.171 (m), 2.560 (m), 2.935 (dd), 2.980 (dd), 3.782 (m), 4.572 (dd)	–	–	–	↓	–	↓	–
Succinate HMDB0000254	2.406 (s)	–	–	–	↓	↓	↓	–
Aspartate HMDB0000191	2.684 (dd), 2.816 (dd), 3.902 (dd)	↓	↓	↓	↑	↑	–	↑
Creatine HMDB0000064	3.041 (s), 3.931 (s)	–	–	–	–	–	↓	–
Creatine phosphate HMDB0001511	3.045 (s), 3.95 (s)	↓	–	–	–	↑	↑	↓
Choline HMDB0000097	3.205 (s)	–	–	–	↑	–	↓	↑
Phosphoryl-choline HMDB0001565	3.224 (s), 3.597 (s), 4.171 (m)	↓	↓	–	–	–	–	↓
Glycerophospho-choline HMDB00086	3.234 (s), 4.33 (m)	↑	–	–	↓	↓	↓	↓
Taurine HMDB0000251	3.269 (t), 3.425 (t)	↓	–	–	–	↑	↓	↑
Myo-inositol HMDB0000211	3.284 (t), 3.540 (dd), 3.626 (dd), 4.067 (t)	↑	↓	–	↑	–	–	–
Inosine HMDB0000195	3.845 (dd), 3.915 (dd), 4.282 (m), 4.442 (dd), 6.105 (d), 8.241 (s), 8.348 (s)	↑	–	↑	↑	↑	↑	↑
Glucose HMDB0000122	4.652 (d), 5.239 (d)	↑	↑	–	–	–	–	–
Uridine HMDB0000296	5.907 (d), 5.922 (d), 7.877 (d)	↑	–	↑	↑	↑	↑	↑
GTP HMDB0001273	5.945 (d), 8.14 (s)	↓	↓	–	↓	↓	↓	↓
UMP HMDB0000288	5.97 (d), 8.11 (d)	↓	–	–	↓	↓	↓	–
NAD HMDB0000902	6.044 (d), 6.09 (d), 8.18 (s), 8.20, 8.43 (s), 8.84 (m), 9.15, 9.34	–	–	–	–	↓	↓	–
Fumarate HMDB00134	6.521 (s)	↓	–	–	↑	–	–	–
Tyrosine HMDB00158	6.91 (m), 7.20 (m)	↓	–	↓	↓	↓	↓	–
Phenylalanine HMDB00159	7.34 (d), 7.381 (m), 7.43 (m)	↓	–	↓	↓	↓	↓	–
AMP HMDB00045	8.273 (s), 8.614 (s)	↓	–	↓	↓	↓	↓	↓
ATP HMDB00538	8.275 (s), 8.538 (s)	↓	–	↓	↓	↓	↓	–

**Table 2:** Intracellular metabolites discriminating between *KRAS*^*G13D/*+^ and *KRAS*^+*/*+^

Metabolite^a^	ppm	p-values	q-values	Log_2_ FC
Leucine	0.965	9.83E−03	7.24E−02	− 0.352
Isoleucine	1.009	3.20E−03	3.62E−02	− 0.405
Valine	1.041	3.68E−03	3.93E−02	− 0.403
Glutamate	2.340	8.67E−03	6.67E−02	− 0.382
Glutamine	2.440	0.010368	0.075063	− 0.167
Aspartate	2.704	1.25E−03	2.12E−02	− 0.412
Creatine phosphate	3.045	1.44E−02	9.28E-−02	− 1.060
Phosphocholine	3.224	3.76E−04	1.09E−02	− 0.733
Glycerophosphocholine	3.234	1.07E−03	1.94E−02	0.590
Taurine	3.428	1.16E−03	2.04E−02	− 0.776
Myo-inositol	4.067	5.05E−04	1.28E−02	0.681
AMP	4.518	8.57E−04	1.74E−02	− 0.705
Glucose	5.235	1.26E−02	8.52E−02	0.646
UDP-*N*-acetylglucosamine	5.512	2.56E−05	3.28E−03	0.659
UDP-*N*-acetylgalactosamine	5.546	9.92E−06	2.93E−03	0.375
Uridine	5.900	6.47E−03	5.54E−02	0.284
Fumarate	6.521	9.21E−04	1.80E−02	− 0.281
Tyrosine	6.899	5.57E−03	5.06E−02	− 0.163
Phenylalanine	7.329	5.17E−03	4.84E−02	− 0.177
UMP	8.105	1.61E−03	2.47E−02	− 0.483
GTP	8.140	5.87E−04	1.40E−02	− 0.238
Inosine	8.349	2.25E−05	3.24E−03	1.739
ATP	8.537	1.87E−03	2.68E−02	− 0.363
AMP	8.605	1.53E−03	2.39E−02	− 1.435

^a^For HMDB ID numbers, see Table 1 above

*FC* fold change

**Fig. 3:**
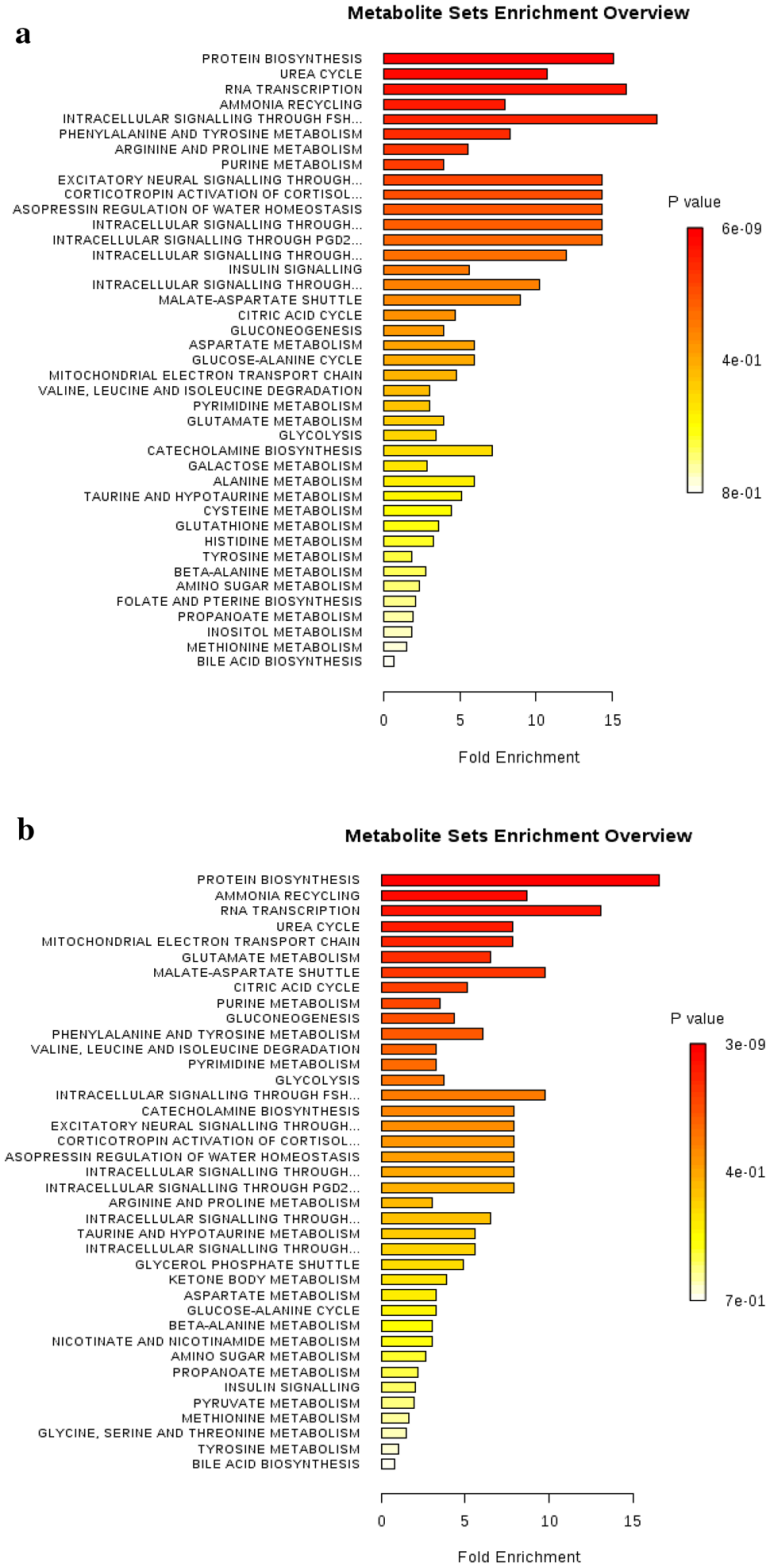
Metabolic pathway analyses for metabolites discriminating between **a** KRAS^G13D/+^and KRAS^+/+^**b** KRAS^G12D/+^ and KRAS^+/+^. The horizontal bars summarize the main metabolite sets identified in this analysis; the bars are coloured based on their p-values and the length is based on the fold enrichment

To further understand the biological significance of the metabolite changes in the *KRAS* mutant clones, we used enrichment analysis (EA) tools in MetaboAnalyst to link metabolites to metabolic pathways (Fig. 3 and Supplementary Figs. 10–12). In all seven *KRAS* mutant clones, protein biosynthesis, urea cycle, RNA transcription and ammonia recycling pathways were over-represented. The levels of amino acids involved in protein synthesis were mostly lower in the mutant *KRAS* clones compared to WT, with the exception of the greater amount of aspartate in the G12C, G12D and G12R clones (Supplementary Table 9). All G12 *KRAS* mutants showed a lower level of glutamine. In parallel, both G13 and G12 mutations (except for G12A, G12D and G12R, where there was no significant change) had a lower intracellular concentration of glutamate, indicating that most *KRAS* mutations used glutaminolysis to provide the energy for growing and proliferation. G12D and G12V mutants also had lower intracellular concentrations of NAD+, a vital coenzyme regulating several cellular metabolic pathways. Moreover, with the exception of KRAS G12C, all cell lines with mutations in codon 12 showed lower levels of UDP-*N*-acetylglucosamine and UDP-*N*-acetylgalactosamine, while KRAS G13D exhibited an increased level of UDP-*N*-acetylglucosamine and UDP-*N*-acetylgalactosamine compared to KRAS WT. All *KRAS* mutants with the exception of KRAS G12S showed lower levels of adenosine monophosphate (AMP) and higher levels of inosine and uridine compared to wild type. Furthermore, lower levels of UMP were observed in all *KRAS* mutants except for G12S, G12A and G12R. Analysis of intracellular metabolites in G12 KRAS mutations revealed decreased level of GPC in cell lines harbouring G12C, G12D, G12V and G12R mutations. Phosphocholine levels were reduced in cell lines with G12R mutation, while the levels of choline were increased in G12C and G12R and decreased in G12V. G13D mutants exhibited significantly higher levels of GPC compared to WT. Choline levels were not found to be significantly different between G13D mutants and WT, while PCho levels were found to be lower in mutated cells. While some metabolites showed similar patterns of changes across the majority of *KRAS* mutants, others were unique for each KRAS mutation. For instance, the levels of taurine were reduced in cell lines with G13D and G12V mutations, whilst increased in cell lines harbouring G12D and G12R mutations. KRAS G13D and G12C also had higher intracellular concentrations of myo-inositol, while G12S exhibited reduced levels of myo-inositol compared to WT.

### Extracellular metabolic profiling of isogenic KRAS wild type and CRC cells with mutation in codon 12 and 13

To better understand metabolite excretion or utilization by cells, we also compared the extracellular metabolite profiles of the SW48 colorectal cell lines with *KRAS* mutation in either codon 12 or 13 with their corresponding KRAS wild type cells. Supplementary Fig. 13 shows representative (1D) ^1^H NMR spectra of culture media (Opti-MEM Medium) from three of these SW48 isogenic cell line (*KRAS*^*G13D/*+^*, KRAS*^*G12D/*+^ and *KRAS*^+*/*+^).

PCA Analysis, showed that all mutant colorectal cancer cell lines were clearly separated from the wild-type *KRAS* controls (Supplementary Fig. 14). Whilst several *KRAS* mutants overlapped with each other, samples of *KRAS* G12D and *KRAS* G12S deviated from the rest of *KRAS* mutants, and *KRAS* G12D clustered closer to the *KRAS* WT.

Maximum margin criterion (MMC, Supplementary Fig. 15) analysis was also performed (Veselkov et al. 2014) and validated (Supplementary Fig. 16) and ANOVA with an FDR of 0.1 identified metabolites responsible for the group separations (Supplementary Figs. 17–23).

Table 3 lists the statistically significant *extracellular* metabolites discriminating between SW48 cell lines harbouring *KRAS* mutations in codon 12 and 13 and their matched wild type cells.

**Table 3:** Summary of the most significant *extracellular* metabolites differentiating between media of SW48 cells with *KRAS* mutations in codons 12 and 13 and media of their wild type counterpart

Metabolite (HMDB no)	^1^H NMR chemical shift in ppm and multi-plicity	*G13D/*+ vs +*/*+	*G12S/*+ vs +*/*+	*G12A/*+ vs +*/*+	*G12C/*+ vs +*/*+	*G12D/*+ vs +*/*+	*G12V/*+ vs +*/*+	*G12R/*+ vs +*/*+
3-methyl-2-oxo-valeric acid (00491)	0.897 (t), 1.102 (d)	↓	–	–	↑	–	↓	↑
keto-leucine (00695)	0.94(d), 2.617(d)	↓	–	↓	–	–	↓	–
Isoleucine (00172)	0.943 (t), 1.01 (d), 3.675 (d)	–	–	–	↓	–	–	↓
Leucine (00687)	0.961 (d), 0.972 (d), 1.691 (m), 1.720 (m), 1.748 (m), 3.737 (dd)	–	–	–	↓	–	–	↓
Valine (00883)	0.996 (d), 1.046 (d), 3.615 (d)	–	–	–	↓	–	–	↓
2-Oxo-isovaleric acid (00019)	1.126 (d)	↓	–	–	–	–	↓	↑
Lactate (00190)	1.331 (d), 4.113 (q)	↓	↓	↑	↑	–	↑	↑
Threonine (00167)	1.335 (d), 3.591 (d)	↓	–	–	–	–	–	–
Alanine (00161)	1.48 (d), 3.787 (q)	–	↓	–	–	↑	↑	–
l-Alanyl-l-glutamine (0028685)	1.546 (d), 1.98 (m), 2.35 (t), 4.182	↓	↑	↓	–	↓	↓	↓
Acetate (00042)	1.919 (s)	↑	↑	↑	↓	–	–	↓
Glutamate (00148)	2.059 (m), 2.140 (m), 2.355 (m), 3.761 (dd)	↑	–	–	–	–	↑	↑
Glutamine (00641)	2.145 (m), 2.46 (m), 3.783 (t)	–	↓	–	–	↑	–	–
Methionine (0000696)	2.1 (s), 2.65 (t)	↓	↓	↓	↓	–	↓	↓
Pyruvate (0000243)	2.379 (s)	–	–	–	–	↑	↑	–
Aspartate (00191)	2.684 (dd), 2.816 (dd), 3.902 (dd)	–	–	–	–	–	–	↓
Glucose (00122)	4.65 (d),5.2 (d)	–	–	↓	↓	–	↓	↓
Histidine (00177)	7.115 (d), 7.922 (d)	–	–	–	–	–	–	↓
Tyrosine (0000158)	6.91 (m),7.20 (m)	–	–	–	–	–	–	↓
Phenyl-alanine (0000159)	7.34 (d), 7.381 (m), 7.43 (m)	↓	–	–	↓	–	↓	↓
Tryptophan (0000929)	7.55 (d), 7.75 (d), 7.29 (s)	↓	–	↓	↓	–	–	↓
Formate (0000142)	8.461 (s)	–	–	–	–	–	↓	↓

Some metabolites showed similar patterns of changes in the majority of the *KRAS* mutant cells. For instance, all *KRAS* mutants, with the exception of G13D, G12D and G12S, showed an elevated uptake of glucose and release of lactate compared to their matched *KRAS* wild type cells. In addition, an increased uptake of methionine is observed in all *KRAS* mutant cell lines apart from G12D. Moreover, l-alanyl-l-glutamine was decreased across all *KRAS* mutant cell lines except G12C and G12S. However, nutrient utilization generally was varied among colorectal cancer cell lines with different *KRAS* mutations (see Supplementary Information p. 34 and Supplementary Tables 10–16).

### Intracellular metabolic profiling of isogenic KRAS wild type and CRC cells with KRAS mutations in codon 61 and 146

Representative annotated NMR spectra for each mutant and WT samples are shown in Supplementary Fig. 24. PCA revealed clear separation of each mutant from KRAS WT (Supplementary Fig. 25). Maximum margin criterion analysis was conducted (Supplementary Fig. 26) (Veselkov et al. 2014) and leave-one-out cross-validation with the quadratic as a classifier (Supplementary Fig. 27) gave correct classification rates of 100% for all models. Supplementary Figs. 28–30 show the representative ANOVA built between (*KRAS*^*A146T/*+^ vs *KRAS*^+*/*+^), (*KRAS*^*Q61H/*+^ vs *KRAS*^+*/*+^) and (*KRAS*^*A146T/*+^ vs *KRAS*^*Q61H/*+^) respectively. Supplementary Table 17 lists the most significant metabolites responsible for discriminating between all three genotypes. *KRAS* mutations at codon 61 and 146 mostly downregulated metabolites relative to the WT, as observed in cells harbouring *KRAS* mutations in codon 12 and 13.

Both A146T and Q61H *KRAS* mutations induced alteration of the same metabolic pathways with protein biosynthesis being the most enriched pathways in both mutations (Supplementary Fig. 31 and Supplementary Tables 18 and 19). Interestingly, the least metabolic changes were observed between *KRAS*^*A146T/*+^ and *KRAS*^*Q61H/*+^.

## Discussion

Mutations in the Kirsten Ras (*KRAS*) oncogene occur in 30–40% of colorectal cancers (CRCs) and have been associated with poor prognosis and resistance to anti-epidermal growth factor receptor (EGFR)-targeted cancer therapy (Andreyev et al. 2001; Arrington et al. 2012; Misale et al. 2014, 2012; Phipps et al. 2013; Tougeron et al. 2013) such as the monoclonal antibody panitumumab. Recent evidence indicates that not all mutations in KRAS have the same biological impact and patients with different KRAS mutations at codons 12 and 13 display different responses to treatments (Garassino et al. 2011; Miller and Miller 2011; Stolze et al. 2015; Tural et al. 2013).

The oncogene *KRAS* plays a critical role in metabolic reprogramming and metabolic interventions are now attracting attention as potential therapeutic modalities (Kawada et al. 2017; Kimmelman 2015). Metabonomics can thus be regarded as a promising tool to gain more insight into metabolic deregulation associated with different *KRAS* mutations, which in turn can be used to discover new therapeutic approaches and better direct existing treatments of CRC.

We note that the impact of KRAS on cell growth is only evident when cells are challenged by changing their environment, such that the impact of a dominant oncogene becomes more apparent. The effects of mutant KRAS in adherent cells, such as SW48, growing on tissue culture plastic is not evident (i.e. the cells are not dependent on mutant KRAS for proliferation or survival under these conditions). However, the impact of mutant KRAS is apparent in adherent cells when they are cultured either on soft agar, or in low adherence plates in the absence of foetal calf serum, and hence we selected these conditions. In addition, we selected a short cell incubation time of 6 h, as we wanted to understand the initial impact that mutant KRAS has on metabolism in cells under conditions where mutant KRAS has the most substantial impact.

Following a thorough multivariate and FDR-controlled univariate analysis of the ^1^H NMR spectra, we observed a clear distinction in both the extracellular (media) and intracellular metabolic profiles of the *KRAS* mutant cells versus the *KRAS* wildtype cells.

The analysis of the extracellular media revealed increased glucose consumption and lactate release in KRAS G12A, KRAS G12C, KRAS G12V and KRAS G12R compared to KRAS wild type cell lines. These metabolic changes could indicate enhanced glycolytic flux in these KRAS mutants. This is consistent with the Warburg effect(Vander Heiden et al. 2009; Warburg 1956) and in accordance with previous studies reporting enhanced glucose uptake in cells with mutant *KRAS* through increased expression of the glucose transporter GLUT1, which subsequently results in increased glycolysis and lactate production (Bryant et al. 2014; Kawada et al. 2017; Yun et al. 2009). Interestingly, lower levels of lactate were observed in the extracellular media of *KRAS* G13D and *KRAS* G12S and intracellular extracts of *KRAS* G12D compared to their wild type counterpart. This implies that glycolytic flux and lactate production are affected by specific mutations in KRAS. It is already known that a glycolytic switch occurs in *KRAS *^*G12D/G12D*^ cells (Kerr et al. 2016).

Decreased levels i.e. elevated consumption of extracellular methionine were observed in all *KRAS* mutant cell lines, with the exception of G12D, relative to their *KRAS* wild type counterpart. Methionine is a sulfur-containing, essential amino acid that plays a critical role in many biological functions within carcinogenic processes. Methionine is involved in cellular processes and is the immediate precursor of S-adenosyl methionine (SAMe), which is a substrate for polyamine synthesis and a key metabolite in one-carbon metabolism, serving as a major methyl donor in a multitude of cellular methylation reactions, including the methylation of cytosine bases on DNA (Cavuoto and Fenech 2012). Aberrations in DNA methylation have been shown to be associated with colorectal carcinogenesis (Kim 2004). Many cancer-cell types exhibit an increased requirement for methionine (Vazquez et al. 2016). Thus, methionine restriction can potentially be exploited to treat cancers that exhibit methionine dependency, as diet is known to influence methionine metabolism in addition to methionine pathway mutations (Sanderson et al. 2019a; b).

Decreased levels of the media component l-alanyl-l-glutamine (stable and non-toxic glutamine source) were found for colorectal cancer cells harbouring G13D, G12A, G12D, G12V and G12R mutations compared to their background-matched *KRAS* wild type cancer cells. No trace of l-alanyl-l-glutamine was observed in the intracellular matrix indicating either that l-alanyl-l-glutamine is hydrolysed by extracellular peptidase activity, or if it is imported, it remains at low levels undetectable by NMR spectroscopy.

Lowered levels of media l-alanyl-l-glutamine with no changes in extracellular glutamine in *KRAS* G13D, G12A, G12V and G12R relative to WT *KRAS* could indicate an elevated glutamine uptake. KRAS G12D also showed significant decrease of l-alanyl-l-glutamine (fold change of ca. 0.16) but with a minor increase of extracellular glutamine (fold change of ca. 1.13) compared to WT *KRAS,* also indicating enhanced glutamine uptake. KRAS G12C, however, displayed no changes in either l-alanyl-l-glutamine or extracellular glutamine compared to WT *KRAS*, which is interesting as this specific KRAS mutation is now targeted with drugs in the clinic.(Canon et al. 2019; Hallin et al. 2019; Seton-Rogers. 2019) Finally, *KRAS* G12S showed less glutamine uptake as suggested by higher levels of l-alanyl-l-glutamine and lower levels of extracellular glutamine indicating less hydrolysis of the dipeptide. Notably, all *KRAS* mutants, with the exception of *KRAS* G13D, showed lower intracellular levels of glutamine indicating increased glutamine consumption.

Glutamine is one of the most important nutrients that is metabolised by many human cancer cell lines. Despite being a non-essential amino acid (NEAA) that can be synthesized de novo*,* many cancer cells in vitro largely depend on exogenous glutamine for their growth, proliferation, and survival (Choi and Park 2018). Once imported into the cells, glutamine provides a carbon source to fuel the tricarboxylic acid (TCA) cycle and a nitrogen source for the biosynthesis of nucleotides, NEAA and hexosamines (Kawada et al. 2017). Cancer cells, particularly those harbouring oncogenic *KRAS*, have been shown to be heavily dependent on glutamine for survival and proliferation (Choi and Park 2018). Overall, our observations suggest that *KRAS* mutations generally induce glutamine dependency with different mutations showing different extents of dependency on extracellular glutamine.

Analysis of the intracellular metabolic profile of the isogenic cell lines also showed significant variation in a number of metabolic pathways between WT *KRAS*-expressing cells and all the other mutants. The main alterations associated with mutant *KRAS* involved changes in amino acid and nucleotide metabolism as well as in the hexosamine biosynthesis pathway.

Intracellular glutamate levels were decreased in most KRAS mutant colorectal cancer cells (KRAS G13D, KRAS G12S, KRAS G12C and KRAS G12V). Intracellular glutamate is primarily a product of glutamine, which is produced by the action of glutaminase (Kawada et al. 2017). Low intracellular levels of glutamine and glutamate in *KRAS* mutant cells compared to their isogenic wildtype cell lines could indicate sustained biosynthetic reactions and a prominent role of glutaminolysis.

Interestingly, we also observed enhanced intracellular metabolism of other amino acids such as tyrosine, phenylalanine, and the branched chain amino acids (BCAAs) leucine, isoleucine, and valine in all KRAS mutants, with the exception of G12S and G12R, compared to their wild type counterpart. BCAAs are essential for cancer growth and are used by cancer cells in various biosynthetic pathways and as a source of energy (Ananieva and Wilkinson 2018). Previous studies have highlighted that leucine, isoleucine, valine, tyrosine and phenylalanine contribute to the TCA cycle via conversion into succinate and fumarate (Owen et al. 2002; Tripathi et al. 2012). Mayers et al. reported that elevated patient plasma BCAA levels were associated with an increased risk of future pancreatic cancer (Mayers et al. 2014). They also reported that changes in BCAA levels in *KRAS*-driven tumours in mice were tissue specific (Mayers et al. 2016).

All KRAS mutants except G12C showed decreased intracellular levels of UDP-GlcNAc while *KRAS* G13D exhibited an increased amount of UDP-GlcNAc relative to the wild type. UDP-GalNAc was also decreased in all *KRAS* mutants except G12C, A146T and Q61H compared to the WT counterpart while G13D showed increased levels. Cancer cells generally exhibit upregulation of the hexosamine biosynthetic pathway (HBP), which is in turn linked to aberrant *O*-GlcNAcylation and an enhanced malignant phenotype (Ferrer et al. 2014; Wong et al. 2017; Yi et al. 2012). Hyper *O*-GlcNAcylation has been reported in PDAC and this modification has been suggested to aid evading of apoptosis (Bryant et al. 2014). Kucharzewska et al. (2015) reported decreased UDP-Gal and UDP-GlcNAc/UDP-GalNAc in hypoxic cancer cells, indicating increased demand of for the synthesis of glycoproteins and glycolipids. In the present study, alterations in the concentrations of UDP-GlcNAc and UDP-GalNAc in almost all of *KRAS* mutants relative to their wild type counterparts could indicate changes in protein glycosylation and HBP synthesis pathway.

Compared to *KRAS* WT, all *KRAS* mutants except G12S demonstrated increased levels of inosine and uridine together with decreased levels of AMP, whilst cell lines with the KRAS A146T mutations showed no such changes and KRAS Q61H just exhibited decreased levels of AMP. The levels of ATP, GTP and UMP were also reduced in most KRAS mutant colorectal cancer cells compared to their corresponding wild type. However, in direct contrast, KRAS A146T displayed increased levels of GTP and ATP. Nucleotide pool imbalance has been proposed to be associated with enhanced mutagenesis and genomic instability, which promotes cancer (Kohnken et al. 2015; Papadopoulou et al. 2015). Deregulation of nucleotide metabolism is associated with a wide range of pathological conditions including cancer (Buj and Aird 2018; Kohnken et al. 2015). In the present study, the significant increase of nucleosides (inosine, uridine) together with decrease of nucleotides (AMP, UMP) in the KRAS mutants could reflect rapid nucleotide turnover and salvage pathway activity of nucleic acid metabolism.

## Conclusion

We carried out a comprehensive, unbiased metabonomic analysis of human colorectal cell lines (SW48) harbouring a wide range of specific *KRAS* mutations on common genetic backgrounds to broaden and deepen our understanding of the relationships between specific genotypes and metabolic phenotypes, or metabotypes, in human colorectal cancer cells. Our data further support the notion that *KRAS* mutations trigger metabolic adaptations to help growth and counter stress. Furthermore, this study highlights that colorectal cancer cells carrying different *KRAS* mutations exhibit specific metabolic phenotypes, including differences in glycolysis, glutamine utilization, and amino acid, choline and nucleotide hexosamine metabolism. These metabolic differences between different *KRAS* mutants might play a role in their different responses to anticancer treatments and hence could be investigated to discover novel metabolic vulnerabilities which might be of utility in the development of more effective targeted therapies against oncogenic *KRAS.* Future studies could also investigate the relative roles of genetic mutation and nutrient availability in directing the metabolic phenotypes of specific cancer cells.

## Electronic supplementary material

Below is the link to the electronic supplementary material.

Supplementary file1 (DOCX 8535 kb)

## Data Availability

Original NMR data are deposited in MetaboLights, EBI, Cambridge UK following publication: https://www.ebi.ac.uk/metabolights/. These data have the code MTBLS1620 (Kenneth Haug, Keeva Cochrane, Venkata Chandrasekhar Nainala, Mark Williams, Jiakang Chang, Kalai Vanii Jayaseelan, Claire O’Donovan. MetaboLights: a resource evolving in response to the needs of its scientific community. Nucleic Acids Research, gkz1019, 10.1093/nar/gkz1019, PMID:31691833).
