# Supplementary material for: Metabolic characterization of colorectal cancer cells harbouring different KRAS mutations in codon 12, 13, 61 and 146 using human SW48 isogenic cell lines
Source: Metabolomics. 2020 Apr 16;16(4):51. doi: 10.1007/s11306-020-01674-2 (PMC7162829; doi:10.1007/s11306-020-01674-2)
Supplement: Supplementary file 1 — Supplementary file1 (DOCX 8535 kb) [file 11306_2020_1674_MOESM1_ESM.docx]

# Supplementary Information: Metabolic characterization of colorectal cancer cells harbouring different *KRAS* mutations in codon 12, 13, 61 and 146 using human SW48 isogenic cell lines

Authors: Dorna Varshavi^a^, Dorsa Varshavi^a^, Nicola McCarthy^b^, Kirill Veselkov^c^, Hector C Keun^d^ and Jeremy R. Everett^a*^

^a^ Medway Metabonomics Research Group,

University of Greenwich,

Chatham Maritime,

Kent,

ME4 4TB

United Kingdom

^b^ Horizon Discovery Ltd.,

Cambridge Research Park,

8100 Beach Dr, Waterbeach,

Cambridge,

CB25 9TL

United Kingdom

^c^ Department of Surgery and Cancer,

Faculty of Medicine,

Imperial College,

London,

SW7 2AZ

United Kingdom

^d^ Department of Surgery and Cancer,

Imperial College London,

Hammersmith Hospital Campus,

London,

W12 ONN UK

United Kingdom

* Author for Correspondence

ORCID: 0000-0003-1550-4482

Telephone: +44 (0)208 331 8323

Email: [j.r.everett@greenwich.ac.uk](mailto:j.r.everett@greenwich.ac.uk)

**Supplementary Table 1.** Data acquisition and processing parameters for 2D NMR spectra of SW48 *KRAS* ^G13D/+^

| **Parameter** | **JRES** | **COSY** | **HSQC** | **HMBC** |
| --- | --- | --- | --- | --- |
| F2 spectral width in Hz | 10,026.7 | 7,211 | 7,211 | 9,615 |
| F1 spectral width in Hz | 78.0 | 7,211 | 28,678 | 34,716 |
| data points in t2 | 8,192 | 4,096 | 2,048 | 2,048 |
| spectral size in F2 | 1,638 |  | 4,096 | 2,048 |
| increments in t1 | 40 | 256 | 400 | 256 |
| spectral size in F1 | 256 | 2,048 | 1,024 | 1,024 |
| number of scans | 16 | 32 | 128 | 50 |
| relaxation delay in seconds | 2.00 | 2.00 | 3.00 | 3.00 |
| apodisation | sine bell in t1 and t2 with first point correction in t1 | sine bell squared in t2, sine square with first point correction in t1 | sine bell squared in t2, sine square with first point correction in t1 | sine bell in t1 and t2 with first point correction in t1 |
| Bruker pulse sequence | jresgpprqf | Cosygpprqf | hsqcetgpprsisp 2.2.be | hmbcgplpndprqf |
| notes | the spectrum was tilted by 45^0^ and symmetrised | t1 noise reduction was applied to the spectrum |  |  |

**Supplementary Table 2.** Data acquisition and processing parameters for 2D NMR spectra of media of SW48 *KRAS* ^+/+^

| **Parameter** | **JRES** | **COSY** | **HSQC** |
| --- | --- | --- | --- |
| F2 spectral width in Hz | 10,026.7 | 9,578 | 9,578 |
| F1 spectral width in Hz | 78.0 | 9,578 | 34,720 |
| data points in t2 | 8,192 | 8,192 | 3,072 |
| spectral size in F2 | 1,638 | 8,192 | 4,096 |
| increments in t1 | 40 | 512 | 800 |
| spectral size in F1 | 256 | 2,048 | 1,024 |
| number of scans | 8 | 12 | 20 |
| relaxation delay in seconds | 2.00 | 2.00 | 3.00 |
| apodisation | sine bell in t1 and t2 with first point correction in t1 | sine bell squared in t2, sine square with first point correction in t1 | sine bell squared in t2, sine square with first point correction in t1 |
| Bruker pulse sequence code | jresgpprqf | Cosygpprqf | hsqcetgpprsisp 2.2.be |
| notes | the spectrum was tilted by 45^0^ and symmetrised | t1 noise reduction was applied to the spectrum |  |

**Supplementary Figure 1.** a) The aliphatic region of the 600 MHz ^1^H NMR spectra from SW48 *KRAS* ^G13D/+,^ *KRAS* ^+/+,^ *KRAS* ^G12D/+^ extracts. Numbers indicate signals corresponding to individual metabolites.1.isoleucine; 2. leucine; 3.valine; 4. lactate; 5. alanine; 6. acetate; 7.glutamate; 8. UDP-*N*-acetylglucosamine;9. UDP-*N*-acetylgalactosamine; 10.glutamine; 11. glutathione; 12.succinate; 13. aspartate; 14.creatine; 15. creatine phosphate; 16. choline; 17. phosphorylcholine; 18. glycerophosphocholine; 19. betaine; 20.taurine; 21. myo-inositol; 22.glycine; 23.glucose

**Supplementary Figure 1.** b) The aromatic region of the 600 MHz ^1^H NMR spectra from SW48 *KRAS* ^G13D/+,^ *KRAS* ^+/+,^ *KRAS* ^G12D/+^ extracts. Numbers indicate signals corresponding to individual metabolites. 8. UDP-*N*-acetylglucosamine; 9. UDP-*N*-acetylgalactosamine; 23. glucose; 24. inosine; 25. NAD; 26. UDP-glucose; 27. AMP; 28. tyrosine; 29. phenylalanine; 30. uridine; 31. ATP


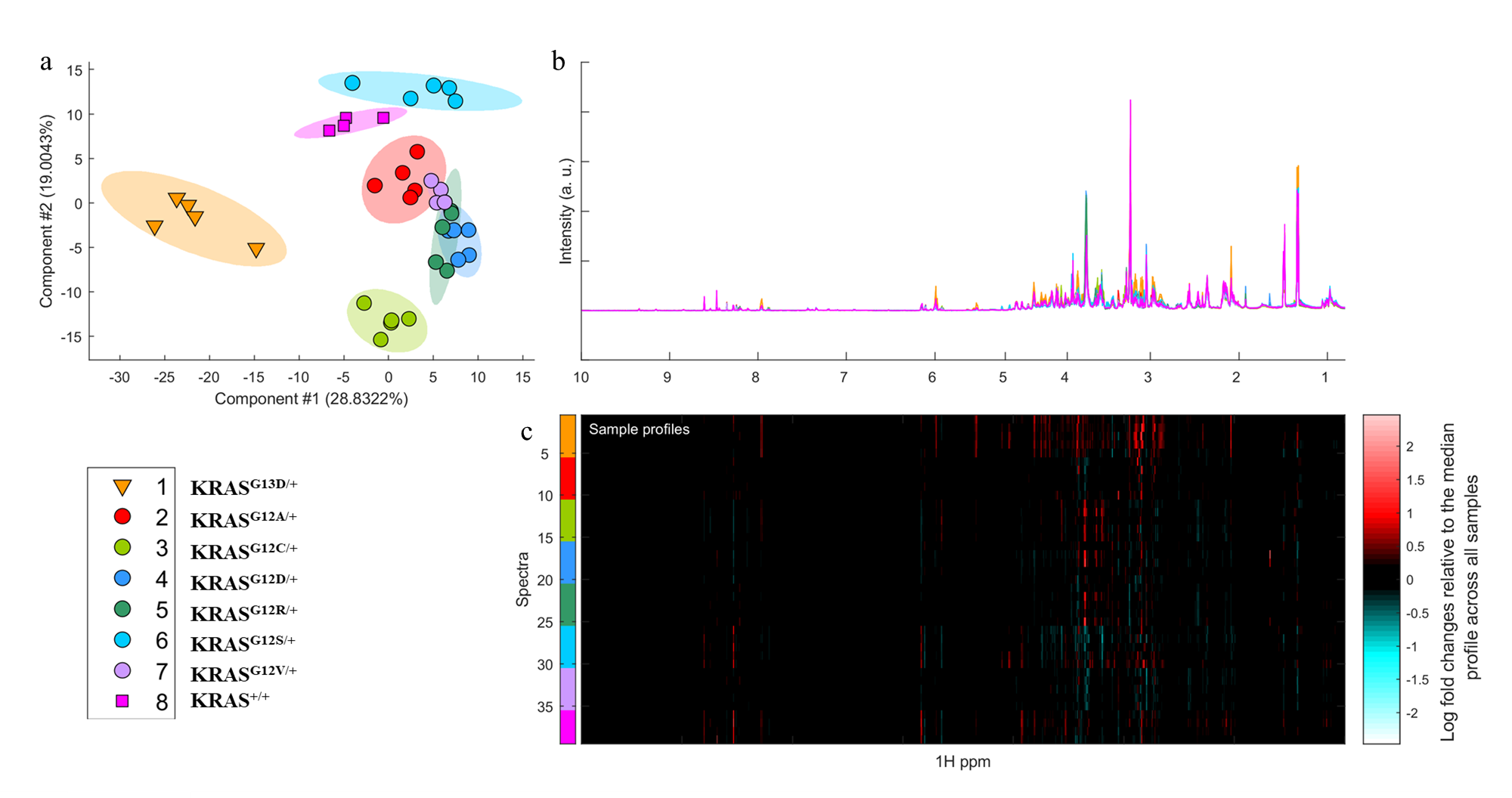


**Supplementary Figure 2.** a) MMC score plot of the 600MHz ^1^NMR spectra of extracts of SW48 cells with *KRAS* mutations in codons 12 and 13 and their wild type counterpart, b) superimposed NMR spectra of SW48 cells, with the same colour coding as in the MMC plot; c) the corresponding ‘heat map display of the 600 MHz ^1^H NMR spectra of SW48 cell lines from 0.8 to 10.0 ppm. Red and blue elements in the spectra indicate NMR signals that are more intense, or less intense, respectively, than the median signal intensity for all the samples.


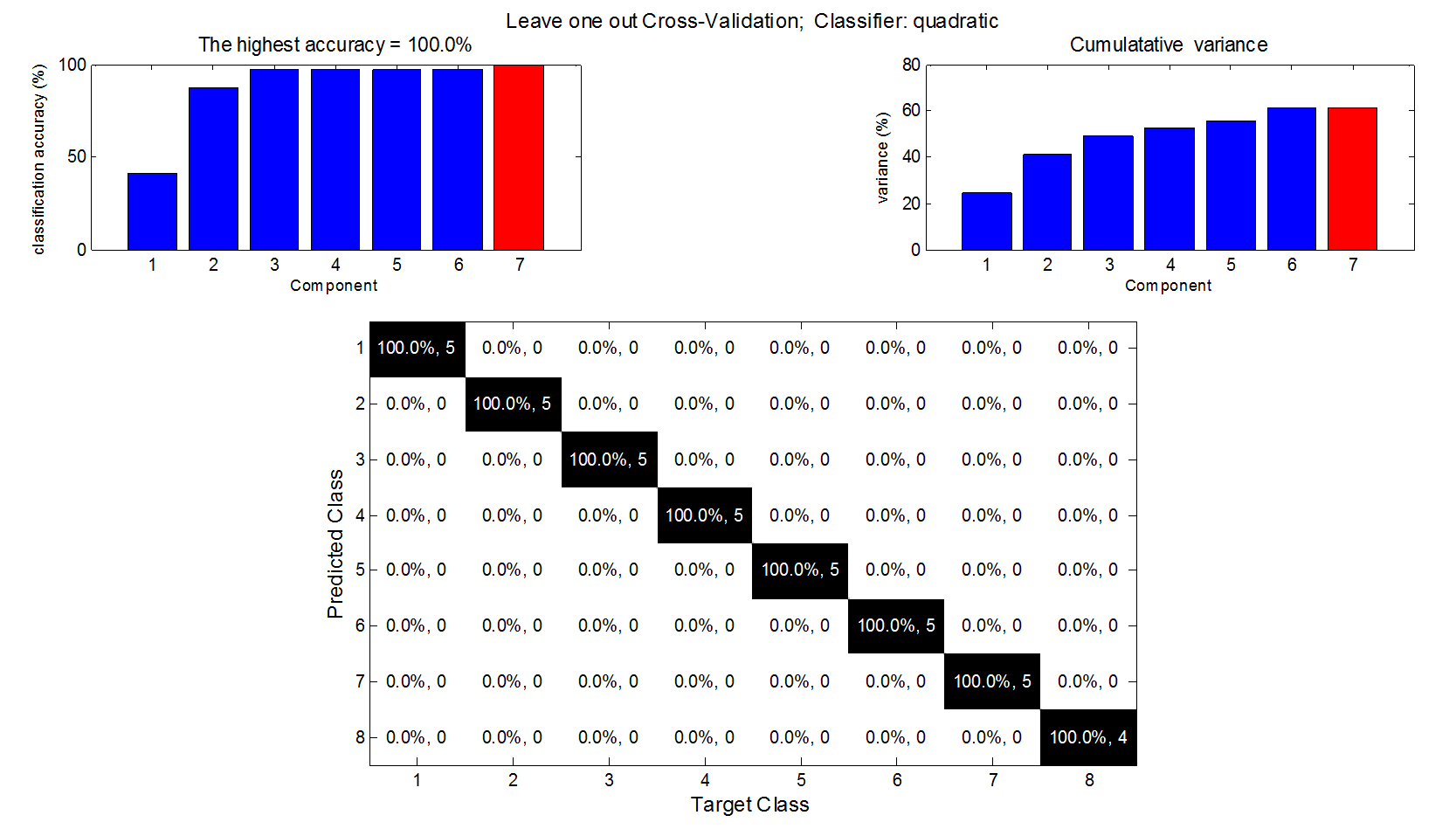


**Supplementary Figure 3.** Leave one out cross validation of the 600 MHz ^1^H NMR spectra from extracts of SW48 cells with *KRAS* mutation at codon 12 and 13 using the quadratic as a classifier; The upper left and upper right diagnostic plots indicate the changes in the percentage of correct classification and the variation related to discrimination as a function of the number of discriminating components respectively. The red color-bar highlights the optimum number of discriminating components. The diagonal of each confusion matrix shows the percentage of samples that are correctly predicted in each class by colouring each box in confusion matrices, black to white with descending predictivity. The off-diagonal boxes represent the percentage of samples in one class being incorrectly predicted into other classes.

**Supplementary Figure 4.** At bottom a ‘heat map display of the 600 MHz ^1^H NMR spectra of *KRAS* ^G12S/+^ (top 5 strips) vs the corresponding spectra of parental cell line (*KRAS*^+/+^) extracts (bottom 4 strips). Red and blue elements in the spectra indicate NMR signals that are more intense, or less intense, respectively, than the median signal intensity for the whole samples. At top, the corresponding ANOVA plot, showing positive peaks for those intracellular metabolite signals that are more intense in *KRAS* ^G12S/+^, and negative peaks for those metabolite signals that are less intense. The signals are colour coded by the p value adjusted for an FDR of 0.1

**Supplementary Figure 5.** At bottom a ‘heat map display of the 600 MHz ^1^H NMR spectra of *KRAS* ^G12A/+^ (top 5 strips) vs the corresponding spectra of *KRAS*^+/+^ extracts (bottom 5 strips). Red and blue elements in the spectra indicate NMR signals that are more intense, or less intense, respectively, than the median signal intensity for the whole samples. At top, the corresponding ANOVA plot, showing positive peaks for those intracellular metabolite signals that are more intense in *KRAS* ^G12A/+^, and negative peaks for those metabolite signals that are less intense. The signals are colour coded by the p value adjusted for an FDR of 0.1.

**Supplementary Figure 6.** At bottom a ‘heat map display of the 600 MHz ^1^H NMR spectra of *KRAS* ^G12C/+^ (top 5 strips) vs the corresponding spectra of parental cell line (*KRAS*^+/+^) extracts (bottom 4 strips). Red and blue elements in the spectra indicate NMR signals that are more intense, or less intense, respectively, than the median signal intensity for the whole samples. At top, the corresponding ANOVA plot, showing positive peaks for those intracellular metabolite signals that are more intense in *KRAS* ^G12C/+^, and negative peaks for those metabolite signals that are less intense. The signals are colour coded by the p value adjusted for an FDR of 0.1.

**Supplementary Figure 7.** At bottom a ‘heat map display of the 600 MHz ^1^H NMR spectra of *KRAS* ^G12D/+^ (top 5 strips) vs the corresponding spectra of parental cell line (*KRAS*^+/+^) extracts (bottom 4 strips). Red and blue elements in the spectra indicate NMR signals that are more intense, or less intense, respectively, than the median signal intensity for the whole samples. At top, the corresponding ANOVA plot, showing positive peaks for those intracellular metabolite signals that are more intense in *KRAS* ^G12D/+^, and negative peaks for those metabolite signals that are less intense. The signals are colour coded by the p value adjusted for an FDR of 0.1.

**Supplementary Figure 8.** At bottom a ‘heat map display of the 600 MHz ^1^H NMR spectra of *KRAS* ^G12V/+^ (top 5 strips) vs the corresponding spectra of parental cell line (*KRAS*^+/+^) extracts (bottom 4 strips). Red and blue elements in the spectra indicate NMR signals that are more intense, or less intense, respectively, than the median signal intensity for the whole samples. At top, the corresponding ANOVA plot, showing positive peaks for those intracellular metabolite signals that are more intense in *KRAS* ^G12V/+^, and negative peaks for those metabolite signals that are less intense. The signals are colour coded by the p value adjusted for an FDR of 0.1.

 **Supplementary Figure 9.** At bottom a ‘heat map display of the 600 MHz ^1^H NMR spectra of *KRAS* ^G12R/+^ (top 5 strips) vs the corresponding spectra of parental cell line (*KRAS*^+/+^) extracts (bottom 4 strips). Red and blue elements in the spectra indicate NMR signals that are more intense, or less intense, respectively, than the median signal intensity for the whole samples. At top, the corresponding ANOVA plot, showing positive peaks for those intracellular metabolite signals that are more intense in *KRAS* ^G12R/+^, and negative peaks for those metabolite signals that are less intense. The signals are colour coded by the p value adjusted for an FDR of 0.1.

**Supplementary Table 3.** Intracellular metabolites discriminating between *KRAS^G12S/+^* and *KRAS^+/+^*

| **Metabolite** | **ppm** | **p-values** | **q-values** | **Log2 FC^1^** |
| --- | --- | --- | --- | --- |
| glutamate | 2.341 | 2.62E-03 | 6.25E-02 | -0.708 |
| glutamine | 2.454 | 4.13E-03 | 8.21E-02 | -0.570 |
| aspartate | 2.705 | 1.50E-03 | 4.57E-02 | -0.365 |
| phosphocholine | 3.224 | 5.43E-03 | 9.75E-02 | -0.697 |
| myo-inositol | 4.067 | 2.57E-03 | 6.18E-02 | -0.629 |
| glucose | 5.235 | 4.93E-02 | 1.00E+00 | 0.515 |
| UDP-*N*-acetylglucosamine | 5.513 | 1.78E-05 | 3.69E-03 | -0.479 |
| UDP-*N*-acetylgalactosamine | 5.546 | 6.43E-05 | 6.94E-03 | -0.197 |
| GTP | 8.14 | 2.40E-04 | 1.63E-02 | -0.231 |

1. FC: fold change

**Supplementary Table 4.** Intracellular metabolites discriminating between *KRAS ^G12A/+^* and *KRAS^+/+^*

| **Metabolite** | **ppm** | **p-values** | **q-values** | **Log 2 FC^1^** |
| --- | --- | --- | --- | --- |
| leucine | 0.966 | 3.70E-04 | 1.62E-02 | -0.538 |
| isoleucine | 1.009 | 2.24E-03 | 4.62E-02 | -0.475 |
| valine | 1.040 | 1.56E-03 | 3.77E-02 | -0.495 |
| threonine | 1.328 | 3.17E-03 | 5.77E-02 | -0.351 |
| glutamine | 2.440 | 3.73E-03 | 6.41E-02 | -0.572 |
| aspartate | 2.704 | 2.33E-03 | 4.72E-02 | -0.370 |
| UDP-*N*-acetylglucosamine | 5.512 | 3.88E-05 | 5.99E-03 | -0.424 |
| UDP-*N*-acetylgalactosamine | 5.546 | 4.49E-04 | 1.79E-02 | -0.165 |
| uridine | 5.900 | 7.48E-04 | 2.42E-02 | 0.464 |
| tyrosine | 6.899 | 4.14E-03 | 6.84E-02 | -0.172 |
| phenylalanine | 7.329 | 3.74E-04 | 1.63E-02 | -0.268 |
| inosine | 8.349 | 3.15E-06 | 2.65E-03 | 1.783 |
| ATP | 8.537 | 2.03E-03 | 4.39E-02 | -0.297 |
| AMP | 8.605 | 1.70E-04 | 1.10E-02 | -1.399 |

1. FC: fold change

**Supplementary Table 5.** Intracellular metabolites discriminating between *KRAS ^G12C/+^* and *KRAS^+/+^*

| **Metabolite** | **ppm** | **p-values** | **q-values** | **Log2 FC^1^** |
| --- | --- | --- | --- | --- |
| leucine | 0.966 | 5.75E-04 | 7.62E-03 | -0.518 |
| isoleucine | 1.009 | 1.60E-03 | 1.60E-02 | -0.462 |
| valine | 1.041 | 1.82E-03 | 1.76E-02 | -0.468 |
| threonine | 1.329 | 6.31E-04 | 8.12E-03 | -0.451 |
| alanine | 1.479 | 5.07E-03 | 3.71E-02 | -0.700 |
| glutamate | 2.341 | 1.09E-02 | 6.43E-02 | -0.360 |
| succinate | 2.406 | 8.40E-04 | 1.00E-02 | -0.902 |
| glutamine | 2.44 | 2.86E-05 | 1.11E-03 | -1.215 |
| glutathione | 2.545 | 1.90E-02 | 9.54E-02 | -0.332 |
| aspartate | 2.705 | 4.55E-03 | 3.43E-02 | 0.408 |
| choline | 3.208 | 2.78E-03 | 2.39E-02 | 0.406 |
| glycerophosphocholine | 3.234 | 4.49E-04 | 6.47E-03 | -0.719 |
| myo-inositol | 4.067 | 2.44E-05 | 1.01E-03 | 1.047 |
| uridine | 5.9 | 1.19E-06 | 2.06E-04 | 1.107 |
| fumarate | 6.521 | 7.06E-03 | 4.71E-02 | 0.221 |
| tyrosine | 6.899 | 1.03E-02 | 6.15E-02 | -0.127 |
| phenylalanine | 7.329 | 1.35E-03 | 1.40E-02 | -0.210 |
| UMP | 8.105 | 9.27E-06 | 6.03E-04 | -0.977 |
| GTP | 8.14 | 3.66E-05 | 1.28E-03 | -0.328 |
| inosine | 8.349 | 2.79E-07 | 1.09E-04 | 1.716 |
| ATP | 8.537 | 2.60E-05 | 1.05E-03 | -0.574 |
| AMP | 8.606 | 1.80E-07 | 9.45E-05 | -2.852 |

1. FC: fold change

**Supplementary Table 6.** Intracellular metabolites discriminating between *KRAS ^G12D/+^* and *KRAS^+/+^*

| **Metabolite** | **ppm** | **p-values** | **q-values** | **Log2 FC^1^** |
| --- | --- | --- | --- | --- |
| leucine | 0.966 | 5.86E-03 | 5.39E-02 | -0.405 |
| isoleucine | 1.010 | 1.08E-02 | 8.05E-02 | -0.197 |
| valine | 1.041 | 8.57E-03 | 6.93E-02 | -0.412 |
| lactate | 1.325 | 6.46E-03 | 5.74E-02 | -0.711 |
| threonine | 1.329 | 1.04E-02 | 7.88E-02 | -0.380 |
| succinate | 2.406 | 7.69E-05 | 3.04E-03 | -0.978 |
| glutamine | 2.44 | 1.23E-02 | 8.81E-02 | -0.275 |
| aspartate | 2.705 | 1.64E-03 | 2.24E-02 | 0.553 |
| creatine phosphate | 3.045 | 9.02E-04 | 1.51E-02 | 0.973 |
| glycerophosphocholine | 3.234 | 1.00E-03 | 1.62E-02 | -0.717 |
| taurine | 3.428 | 4.51E-03 | 4.51E-02 | 0.445 |
| UDP-*N*-acetylglucosamine | 5.513 | 3.68E-05 | 1.95E-03 | -0.463 |
| UDP-*N*-acetylgalactosamine | 5.546 | 2.68E-04 | 6.62E-03 | -0.180 |
| Uridine | 5.9 | 8.28E-05 | 3.20E-03 | 0.779 |
| NAD | 6.05 | 4.35E-03 | 4.40E-02 | -0.168 |
| tyrosine | 6.899 | 1.16E-02 | 8.44E-02 | -0.140 |
| phenylalanine | 7.329 | 6.29E-03 | 5.64E-02 | -0.191 |
| UMP | 8.105 | 6.37E-05 | 2.68E-03 | -0.816 |
| GTP | 8.14 | 1.55E-04 | 4.73E-03 | -0.260 |
| inosine | 8.349 | 8.90E-07 | 2.59E-04 | 1.853 |
| ATP | 8.537 | 1.38E-04 | 4.37E-03 | -0.479 |
| AMP | 8.606 | 3.27E-07 | 1.82E-04 | -2.610 |

1. FC: fold change

**Supplementary Table 7.** Intracellular metabolites discriminating between *KRAS ^G12V/+^* and *KRAS^+/+^*

| **Metabolite** | **ppm** | **p-values** | **q-values** | **Log2 FC^1^** |
| --- | --- | --- | --- | --- |
| leucine | 0.966 | 7.70E-05 | 3.09E-03 | -0.687 |
| isoleucine | 1.009 | 6.02E-04 | 8.89E-03 | -0.565 |
| valine | 1.041 | 3.32E-04 | 6.47E-03 | -0.631 |
| lactate | 1.325 | 1.03E-03 | 1.22E-02 | -0.997 |
| threonine | 1.329 | 1.96E-05 | 1.58E-03 | -0.886 |
| alanine | 1.479 | 2.48E-03 | 2.14E-02 | -0.816 |
| glutamate | 2.341 | 1.20E-03 | 1.35E-02 | -0.677 |
| succinate | 2.406 | 1.99E-05 | 1.59E-03 | -1.297 |
| glutamine | 2.44 | 2.86E-03 | 2.34E-02 | -0.567 |
| glutathione | 2.545 | 5.64E-04 | 8.60E-03 | -0.572 |
| creatine | 3.040 | 3.13E-04 | 6.25E-03 | -1.240 |
| choline | 3.208 | 2.57E-03 | 2.19E-02 | -0.410 |
| glycerophosphocholine | 3.234 | 7.12E-03 | 4.25E-02 | -0.411 |
| taurine | 3.428 | 1.73E-02 | 7.77E-02 | -0.412 |
| UDP-*N*-acetylglucosamine | 5.513 | 4.93E-04 | 7.92E-03 | -0.318 |
| UDP-*N*-acetylgalactosamine | 5.546 | 8.84E-03 | 4.92E-02 | -0.094 |
| uridine | 5.9 | 3.26E-05 | 2.06E-03 | 0.542 |
| NAD | 6.05 | 2.32E-02 | 9.46E-02 | -0.128 |
| tyrosine | 6.899 | 6.80E-04 | 9.53E-03 | -0.270 |
| phenylalanine | 7.329 | 1.08E-04 | 3.77E-03 | -0.303 |
| UMP | 8.105 | 3.61E-04 | 6.72E-03 | -0.623 |
| GTP | 8.14 | 5.17E-03 | 3.44E-02 | -0.161 |
| inosine | 8.349 | 2.87E-05 | 1.92E-03 | 1.566 |
| ATP | 8.537 | 1.91E-04 | 4.98E-03 | -0.423 |
| AMP | 8.606 | 2.42E-06 | 5.77E-04 | -2.253 |

1. FC: fold change

**Supplementary Table 8.** Intracellular metabolites discriminating between *KRAS ^G12R/+^* and *KRAS^+/+^*

| **Metabolite** | **ppm** | **p-values** | **q-values** | **Log2 FC^1^** |
| --- | --- | --- | --- | --- |
| glutamine | 2.44 | 8.43E-05 | 2.77E-03 | -1.132 |
| aspartate | 2.705 | 1.16E-03 | 1.84E-02 | 0.428 |
| creatine phosphate | 3.045 | 2.62E-06 | 5.76E-04 | -1.619 |
| choline | 3.208 | 9.33E-03 | 8.30E-02 | 0.344 |
| phosphocholine | 3.225 | 4.00E-03 | 4.50E-02 | -0.459 |
| glycerophosphocholine | 3.234 | 1.59E-05 | 1.08E-03 | -1.115 |
| taurine | 3.428 | 9.47E-03 | 8.36E-02 | 0.295 |
| UDP-*N*-acetylglucosamine | 5.513 | 1.77E-05 | 1.14E-03 | -0.483 |
| UDP-*N*-acetylgalactosamine | 5.546 | 5.35E-05 | 2.10E-03 | -0.181 |
| uridine | 5.9 | 2.05E-07 | 2.02E-04 | 0.554 |
| GTP | 8.14 | 8.07E-05 | 2.71E-03 | -0.281 |
| inosine | 8.349 | 1.41E-07 | 1.80E-04 | 1.178 |
| AMP | 8.606 | 2.01E-05 | 1.22E-03 | -1.850 |

1. FC: fold change

a b


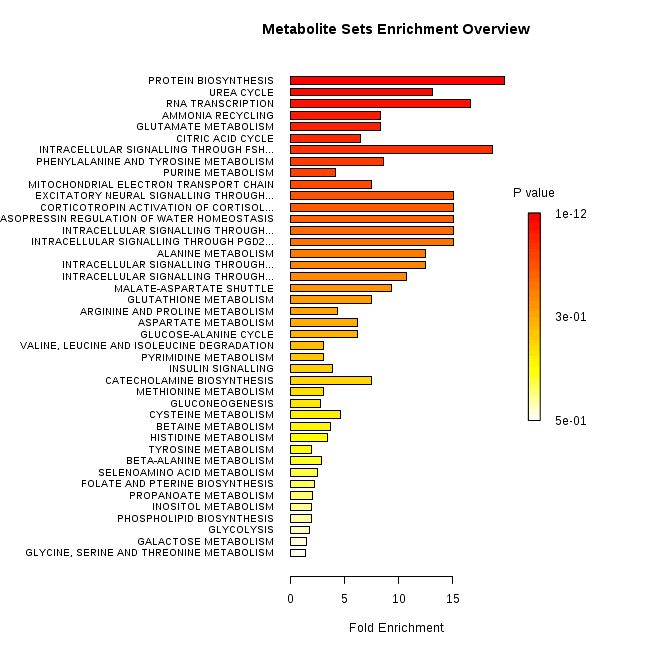

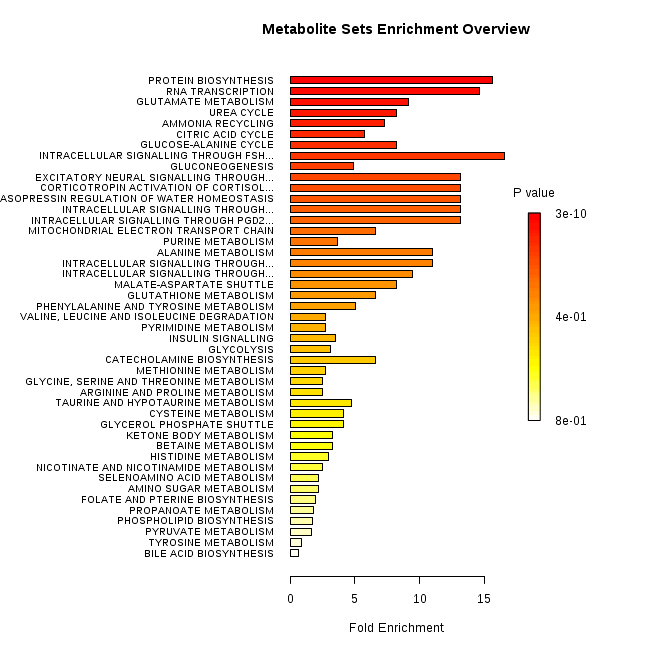


**Supplementary Figure 10.** Metabolic pathway analyses for metabolites discriminating between: a) KRAS ^G12C/+^and KRAS^+/+^ b) KRAS ^G12V/+^and KRAS^+/+^. The horizontal bars summarize the main metabolite sets identified in this analysis; the bars are coloured based on their p-values and the length is based on the fold enrichment

a b


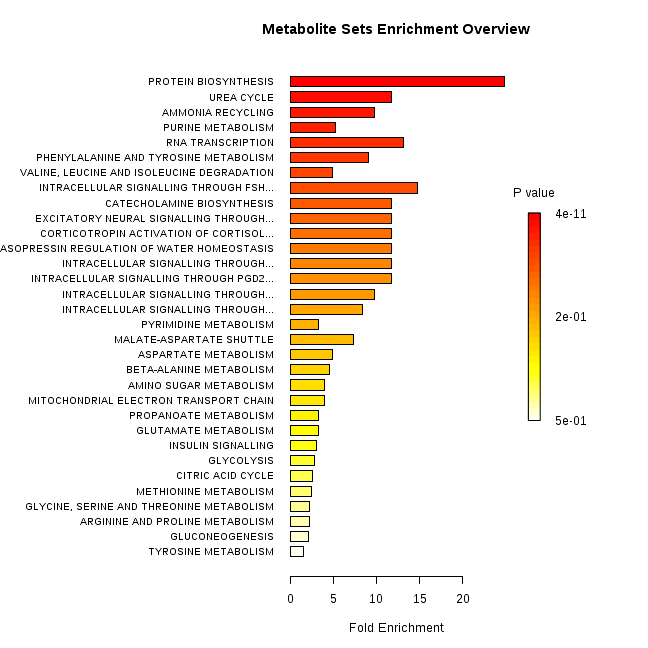

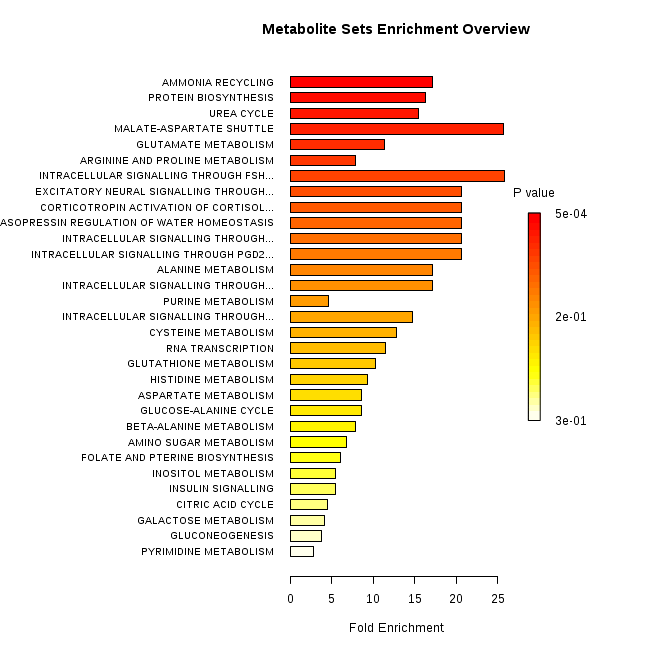


**Supplementary Figure 11.** Metabolic pathway analyses for metabolites discriminating between: a) KRAS ^G12A/+^and KRAS^+/+^ b) KRAS ^G12S/+^and KRAS^+/+^ . The horizontal bars summarize the main metabolite sets identified in this analysis; the bars are coloured based on their p-values and the length is based on the fold enrichment


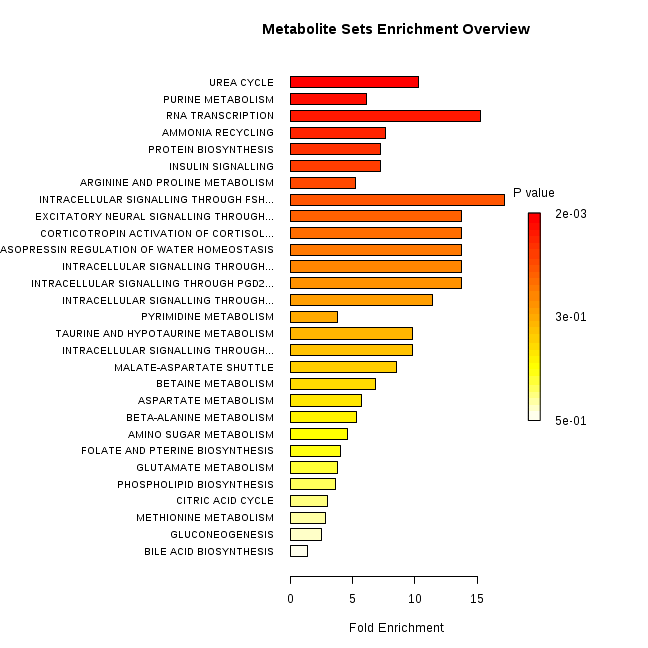


**Supplementary Figure 12.** Metabolic pathway analyses for metabolites discriminating between KRAS ^G12R/+^and KRAS^+/+^. The horizontal bars summarize the main metabolite sets identified in this analysis; the bars are coloured based on their p-values and the length is based on the fold enrichment

**Supplementary Table 9.** Changes in intracellular amino acids involved in protein synthesis

| **Metabolite** | **Chemical shift (ppm)** | \| ***G13D/+***  ***vs +/+*** \|  \|  \| \| --- \| --- \| --- \| | ***G12S/+***  ***vs +/+*** | ***G12A/+***  ***vs +/+*** | ***G12C/+***  ***vs +/+*** | ***G12D/+***  ***vs +/+*** | ***G12V/+***  ***vs +/+*** | ***G12R/+***  ***vs +/+*** |
| --- | --- | --- | --- | --- | --- | --- | --- | --- | --- | --- | --- |
| isoleucine | 0.943 | ⭣ | - | ⭣ | ⭣ | ⭣ | ⭣ | - |
| leucine | 0.961 | ⭣ | - | ⭣ | ⭣ | ⭣ | ⭣ | - |
| valine | 0.996 | ⭣ | - | ⭣ | ⭣ | ⭣ | ⭣ | - |
| threonine | 1.335 | - | - | - | ⭣ | ⭣ | ⭣ | - |
| \| alanine \| \| --- \| | 1.484 | - | - | - | ⭣ | - | ⭣ | - |
| glutamate | 2.059 | ⭣ | ⭣ | - | ⭣ | - | ⭣ | - |
| glutamine | 2.145 | - | ⭣ | ⭣ | ⭣ | ⭣ | ⭣ | ⭣ |
| aspartate | 2.684 | ⭣ | ⭣ | ⭣ | ⭡ | ⭡ | - | ⭡ |
| tyrosine | 6.910 | ⭣ | - | ⭣ | ⭣ | ⭣ | ⭣ | - |
| phenyl-alanine | 7.340 | ⭣ | - | ⭣ | ⭣ | ⭣ | ⭣ | - |

**Supplementary Figure 13.** a) The aliphatic region of the 600 MHz ^1^H NMR spectra from SW48 *KRAS* ^G13D/+,^ *KRAS* ^+/+,^ *KRAS* ^G12D/+^ media. Numbers indicate signals corresponding to individual metabolites. 1. isoleucine; 2. leucine; 3. valine;4. lactate; 5. alanine; 6. L-alanyl-L-glutamine; 7. acetate; 8. glutamine; 9. pyruvate; 10. glucose; 11. threonine

**Supplementary Figure 13.** b) The aromatic region of the 600 MHz ^1^H NMR spectra from SW48 *KRAS* ^G13D/+,^ *KRAS* ^+/+,^ *KRAS* ^G12D/+^ media. Numbers indicate signals corresponding to individual metabolites. 12. tyrosine; 13. histidine; 14. tryptophan; 15. phenylalanine; 16. formate

**Supplementary Figure 14.** a) PCA score plot of the 600MHz ^1^H NMR spectra from media of SW48 cell lines with *KRAS* mutations in codons 12 and 13 and their wild type counterpart, b) superimposed NMR spectra of cell’s media, with the same colour coding as in the PCA plot part a); c) ‘the corresponding heat map display of the 600 MHz ^1^H NMR spectra from 0.8 to 10.0 ppm of media of SW48 cell lines. Red and blue elements in the spectra indicate NMR signals that are more intense, or less intense, respectively, than the median signal intensity for all the samples.


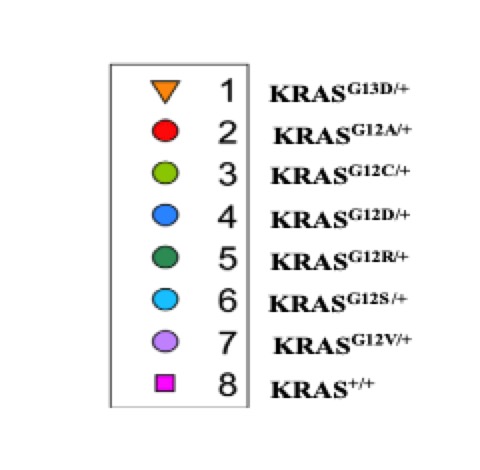


**Supplementary Figure 15.** a) MMC score plot of the 600 MHz ^1^H NMR spectra from media of SW48 cell lines with *KRAS* mutations in codons 12 and 13 and their wild type counterpart, b) superimposed NMR spectra of SW48 cell media, with the same colour coding as in the MMC plot; c) the corresponding ‘heat map display of the 600 MHz ^1^H NMR spectra of Red and blue elements in the spectra indicate NMR signals that are more intense, or less intense, respectively, than the median signal intensity for all the samples.


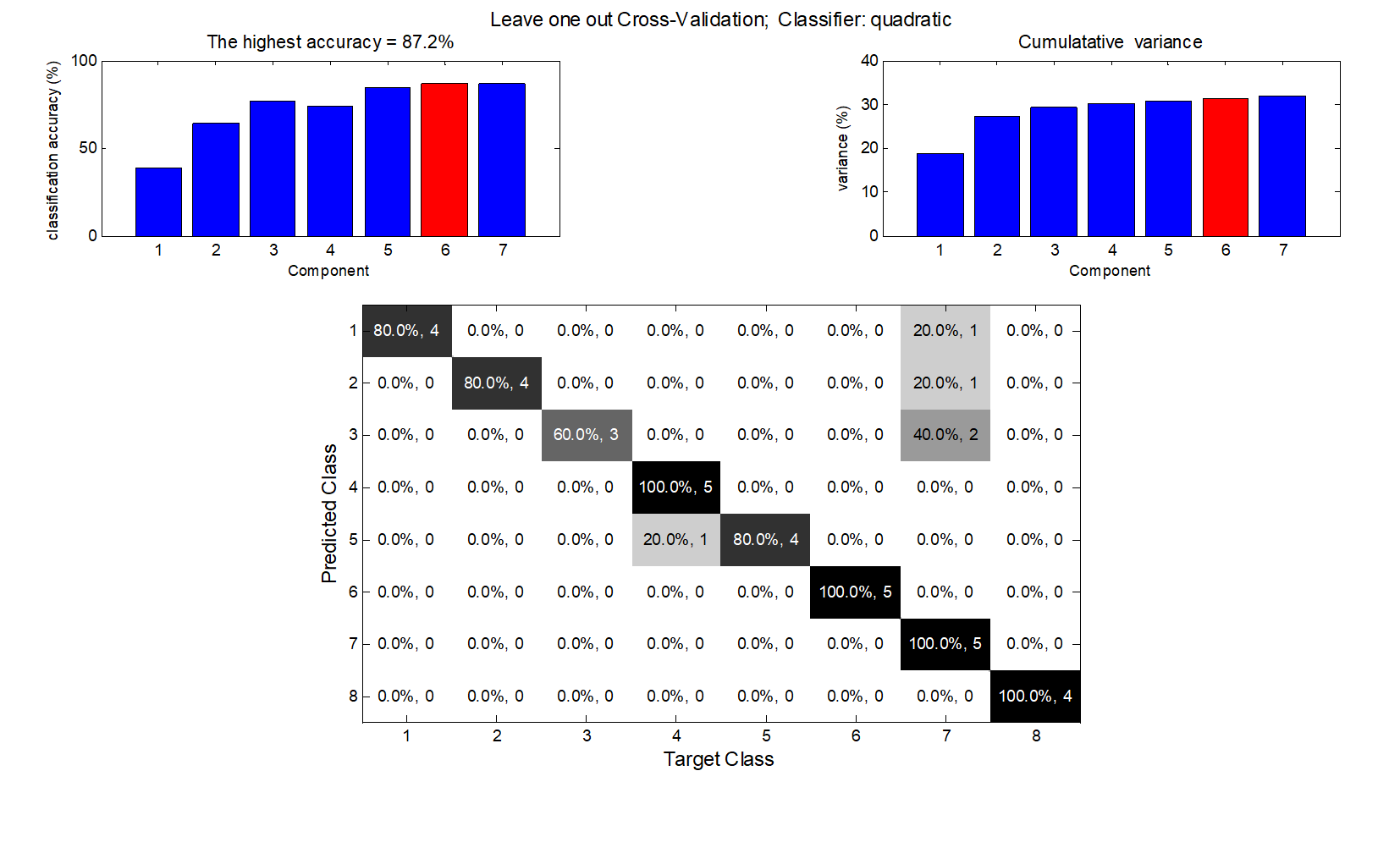


**Supplementary Figure 16.** Leave one out cross validation of the MMC model made from 600 MHz ^1^H NMR spectra of the media of SW48 cell lines with *KRAS* mutation at codon 12 and 13 using the quadratic as a classifier; The upper left and upper right diagnostic plots indicate the changes in the percentage of correct and the variation related to discrimination as a function of the number of discriminating components respectively. The red color-bar highlights the optimum number of discriminating components. The diagonal of each confusion matrix shows the percentage of samples that are correctly predicted in each class by colouring each box in confusion matrices, black to white with a descending predictivity. The off-diagonal boxes represent the percentage of samples in one class being incorrectly predicted into other classes.

 **Supplementary Figure 17.** At bottom a ‘heat map display of the 600 MHz ^1^H NMR spectra from media of *KRAS* ^G13D/+^ (top 5 strips) vs the corresponding spectra from media of *KRAS*^+/+^ (bottom 4 strips). Red and blue elements in the spectra indicate NMR signals that are more intense, or less intense, respectively, than the median signal intensity for the whole samples. At top, the corresponding ANOVA plot, showing positive peaks for those metabolite signals that are more intense in media of *KRAS* ^G13D/+^, and negative peaks for those metabolite signals that are less intense. The signals are colour coded by the p value adjusted for FDR of 0.1.

**Supplementary Figure 18.** At bottom a ‘heat map display of the 600 MHz ^1^H NMR spectra from media of *KRAS* ^G12S/+^ (top 5 strips) vs the corresponding spectra from media of *KRAS*^+/+^ (bottom 4 strips). Red and blue elements in the spectra indicate NMR signals that are more intense, or less intense, respectively, than the median signal intensity for the whole samples. At top, the corresponding ANOVA plot, showing positive peaks for those metabolite signals that are more intense in media of *KRAS* ^G12S/+^, and negative peaks for those metabolite signals that are less intense. The signals are colour coded by the p value adjusted for FDR of 0.1.

 **Supplementary Figure 19.** At bottom a ‘heat map display of the 600 MHz ^1^H NMR spectra from media of *KRAS* ^G12A/+^ (top 5 strips) vs the corresponding spectra from media of *KRAS*^+/+^ (bottom 4 strips). Red and blue elements in the spectra indicate NMR signals that are more intense, or less intense, respectively, than the median signal intensity for the whole samples. At top, the corresponding ANOVA plot, showing positive peaks for those metabolite signals that are more intense in media of *KRAS* ^G12A/+^, and negative peaks for those metabolite signals that are less intense. The signals are colour coded by the p value adjusted for FDR of 0.1.

**Supplementary Figure 20.** At bottom a ‘heat map display of the 600 MHz ^1^H NMR spectra from media of *KRAS* ^G12C/+^ (top 5 strips) vs the corresponding spectra from media of *KRAS*^+/+^ (bottom 4 strips). Red and blue elements in the spectra indicate NMR signals that are more intense, or less intense, respectively, than the median signal intensity for the whole samples. At top, the corresponding ANOVA plot, showing positive peaks for those metabolite signals that are more intense in media of *KRAS* ^G12C/+^, and negative peaks for those metabolite signals that are less intense. The signals are colour coded by the p value adjusted for FDR of 0.1.

**Supplementary Figure 21.** At bottom a ‘heat map display of the 600 MHz ^1^H NMR spectra from media of *KRAS* ^G12D/+^ (top 5 strips) vs the corresponding spectra from media of *KRAS*^+/+^ (bottom 4 strips). Red and blue elements in the spectra indicate NMR signals that are more intense, or less intense, respectively, than the median signal intensity for the whole samples. At top, the corresponding ANOVA plot, showing positive peaks for those metabolite signals that are more intense in *KRAS* ^G12D/+^, and negative peaks for those metabolite signals that are less intense. The signals are colour coded by the p value adjusted for FDR of 0.1.

 **Supplementary Figure 22.** At bottom a ‘heat map display of the 600 MHz ^1^H NMR spectra from media of *KRAS* ^G12V/+^ (top 5 strips) vs the corresponding spectra from media of *KRAS*^+/+^(bottom 4 strips). Red and blue elements in the spectra indicate NMR signals that are more intense, or less intense, respectively, than the median signal intensity for the whole samples. At top, the corresponding ANOVA plot, showing positive peaks for those metabolite signals that are more intense in *KRAS* ^G12V/+^, and negative peaks for those metabolite signals that are less intense. The signals are colour coded by the p value adjusted for FDR of 0.1.

**Supplementary Figure 23.** At bottom a ‘heat map display of the 600 MHz ^1^H NMR spectra from media of *KRAS* ^G12R/+^ (top 5 strips) vs the corresponding spectra from media of *KRAS*^+/+^ (bottom 4 strips). Red and blue elements in the spectra indicate NMR signals that are more intense, or less intense, respectively, than the median signal intensity for the whole samples. At top, the corresponding ANOVA plot, showing positive peaks for those metabolite signals that are more intense in *KRAS* ^G12R/+^, and negative peaks for those metabolite signals that are less intense. The signals are colour coded by the p value adjusted for FDR of 0.1.

**Extracellular Metabolite Profile Differences between KRAS Mutants.**

The media metabolic profile (exometabolome) of *KRAS* ^G13D/+^ showed decreased levels of 3-methyl-2-oxovaleric acid, 2-oxoisovaleric acid, lactate, threonine, L-alanyl-L-glutamine, methionine, ketoleucine, phenylalanine, tryptophan and increased levels of glutamate and acetate (Supplementary Table 10).

Compared to KRAS WT, exometabolome of KRAS G12S was characterised by decreased levels of lactate, alanine, glutamine, methionine and increased levels of L-alanyl-L-glutamine and acetate (Supplementary Table 11).

In KRAS G12A decreased levels of L-alanyl-L-glutamine, methionine, ketoleucine, glucose, tryptophan and increased concentrations of lactate and acetate was observed in comparison to parental cell line (Supplementary Table 12).

KRAS G12C displayed decreased levels of isoleucine, leucine, valine, acetate, methionine, glucose, phenylalanine, tryptophan and higher levels of 3-methyl-2-oxovaleric acid and lactate relative to KRAS WT (Supplementary Table 13).

Compared to WT, KRAS G12D showed decreased levels of L-alanyl-L-glutamine and increased concentrations of alanine, glutamine, pyruvate (Supplementary Table 14).

KRAS G12V exhibited decreased levels of 3-methyl-2-oxovaleric acid, 2-oxoisovaleric acid, L-alanyl-L-glutamine, methionine, ketoleucine, glucose, phenylalanine and formate and increased levels of lactate, alanine, glutamate, pyruvate compared to KRAS WT (Supplementary Table 15).

In KRAS G12R, decreased levels of isoleucine, leucine, valine, L-alanyl-L-glutamine, acetate, methionine, aspartate, histidine, glucose, tyrosine, phenylalanine, tryptophan, formate and increased levels of 3-methyl-2-oxovaleric acid, 2-oxoisovaleric acid, lactate and glutamate were observed in comparison to KRAS WT (Supplementary Table 16).

**Supplementary Table 10.** Extracellular metabolites discriminating between *KRAS^G13D/+^* and *KRAS^+/+^*

| Metabolite | ppm | p-values | q-values | Log 2 FC^1^ |
| --- | --- | --- | --- | --- |
| 3-methyl-2-oxovaleric acid | 1.108 | 1.02E-04 | 2.83E-03 | -0.565 |
| 2-oxoisovaleric acid | 1.121 | 6.28E-05 | 2.13E-03 | -0.516 |
| threonine | 1.330 | 6.33E-03 | 5.06E-02 | -0.324 |
| lactate | 1.338 | 7.30E-04 | 1.06E-02 | -0.220 |
| L-alanyl-L-glutamine | 1.550 | 1.18E-05 | 1.05E-03 | -1.018 |
| acetate | 1.923 | 4.27E-03 | 3.74E-02 | 0.188 |
| glutamate | 2.065 | 4.27E-05 | 1.71E-03 | 0.497 |
| ketoleucine | 2.611 | 4.49E-05 | 1.76E-03 | -0.477 |
| methionine | 2.648 | 9.08E-05 | 2.67E-03 | -0.241 |
| Phenylalanine | 7.342 | 1.03E-02 | 7.20E-02 | -0.106 |
| tryptophan | 7.749 | 6.47E-04 | 9.69E-03 | -0.208 |

1. FC: fold change

**Supplementary Table 11.** Extracellular metabolites discriminating between *KRAS^G12S/+^* and *KRAS^+/+^*

| Metabolite | ppm | p-values | q-values | Log 2 FC^1^ |
| --- | --- | --- | --- | --- |
| lactate | 1.338 | 9.59E-04 | 1.76E-02 | -0.732 |
| alanine | 1.491 | 2.29E-03 | 2.96E-02 | -0.659 |
| L-alanyl-L-glutamine | 1.550 | 8.51E-06 | 2.96E-03 | 1.837 |
| acetate | 1.923 | 2.92E-05 | 2.37E-03 | 0.557 |
| glutamine | 2.453 | 2.90E-03 | 3.43E-02 | -0.661 |
| methionine | 2.648 | 2.16E-03 | 2.86E-02 | -0.134 |

1. FC: fold change

**Supplementary Table 12.** Extracellular metabolites discriminating between *KRAS^G12A/+^* and *KRAS^+/+^*

| Metabolite | ppm | p-values | q-values | Log 2 FC^1^ |
| --- | --- | --- | --- | --- |
| lactate | 1.338 | 1.49E-04 | 3.46E-03 | 0.740 |
| L-alanyl-L-glutamine | 1.550 | 1.13E-03 | 1.34E-02 | -2.490 |
| acetate | 1.923 | 3.27E-03 | 2.92E-02 | 0.308 |
| ketoleucine | 2.611 | 9.09E-03 | 6.23E-02 | -0.253 |
| methionine | 2.648 | 2.24E-03 | 2.20E-02 | -0.259 |
| glucose | 5.242 | 1.75E-03 | 1.84E-02 | -0.225 |
| tryptophan | 7.749 | 6.26E-05 | 2.15E-03 | -0.513 |

1. FC: fold change

**Supplementary Table 13.** Extracellular metabolites discriminating between *KRAS^G12C/+^* and *KRAS^+/+^*

| Metabolite | ppm | p-values | q-values | Log 2 FC^1^ |
| --- | --- | --- | --- | --- |
| leucine | 0.965 | 1.19E-02 | 9.82E-02 | -0.110 |
| isoleucine | 1.007 | 3.08E-03 | 3.71E-02 | -0.175 |
| valine | 1.039 | 1.53E-03 | 2.26E-02 | -0.182 |
| 3-methyl-2-oxovaleric acid | 1.108 | 9.23E-04 | 1.56E-02 | 0.301 |
| acetate | 1.923 | 7.75E-05 | 3.70E-03 | -0.616 |
| lactate | 1.338 | 6.14E-03 | 6.11E-02 | 0.474 |
| methionine | 2.648 | 2.03E-03 | 2.76E-02 | -0.157 |
| glucose | 5.242 | 1.24E-03 | 1.94E-02 | -0.148 |
| phenylalanine | 7.342 | 2.87E-03 | 3.54E-02 | -0.116 |
| tryptophan | 7.749 | 7.01E-03 | 6.73E-02 | -0.152 |

1. FC: fold change

**Supplementary Table 14.** Extracellular metabolites discriminating between *KRAS^G12D/+^* and *KRAS^+/+^*

| Metabolite | ppm | p-values | q-values | Log 2 FC^1^ |
| --- | --- | --- | --- | --- |
| alanine | 1.491 | 3.39E-03 | 7.13E-02 | 0.194 |
| L-alanyl-L-glutamine | 1.550 | 1.28E-03 | 3.87E-02 | -2.680 |
| pyruvate | 2.378 | 2.96E-03 | 6.61E-02 | 0.246 |
| glutamine | 2.453 | 4.90E-03 | 8.98E-02 | 0.176 |

1. FC: fold change

**Supplementary Table 15.** Extracellular metabolites discriminating between *KRAS^G12V/+^* and *KRAS^+/+^*

| Metabolite | ppm | p-values | q-values | Log 2 FC^1^ |
| --- | --- | --- | --- | --- |
| 3-methyl-2-oxovaleric acid | 1.108 | 1.36E-03 | 1.19E-02 | -0.245 |
| 2-oxoisovaleric acid | 1.121 | 1.41E-02 | 7.58E-02 | -0.250 |
| lactate | 1.338 | 1.57E-02 | 8.25E-02 | 0.214 |
| alanine | 1.491 | 1.12E-03 | 1.02E-02 | 0.152 |
| L-alanyl-L-glutamine | 1.550 | 8.99E-04 | 8.57E-03 | -1.204 |
| glutamate | 2.065 | 1.49E-03 | 1.28E-02 | 0.182 |
| pyruvate | 2.378 | 2.39E-03 | 1.85E-02 | 0.358 |
| ketoleucine | 2.611 | 3.40E-03 | 2.45E-02 | -0.232 |
| methionine | 2.648 | 3.85E-05 | 7.54E-04 | -0.194 |
| glucose | 5.242 | 7.23E-04 | 7.34E-03 | -0.094 |
| phenylalanine | 7.342 | 1.34E-03 | 1.17E-02 | -0.098 |
| formate | 8.461 | 7.86E-03 | 4.77E-02 | -0.272 |

1. FC: fold change

**Supplementary Table 16.** Extracellular metabolites discriminating between *KRAS^G12R/+^* and *KRAS^+/+^*

| Metabolite | ppm | p-values | q-values | Log 2 FC^1^ |
| --- | --- | --- | --- | --- |
| leucine | 0.965 | 5.59E-04 | 1.17E-02 | -0.191 |
| isoleucine | 1.007 | 1.44E-03 | 1.71E-02 | -0.256 |
| valine | 1.039 | 1.51E-03 | 1.74E-02 | -0.263 |
| 3-methyl-2-oxovaleric acid | 1.108 | 1.36E-03 | 1.68E-02 | 0.381 |
| 2-oxoisovaleric acid | 1.121 | 2.95E-03 | 2.56E-02 | 0.429 |
| lactate | 1.338 | 5.55E-04 | 1.17E-02 | 0.502 |
| L-alanyl-L-glutamine | 1.550 | 9.31E-03 | 5.36E-02 | -1.323 |
| acetate | 1.923 | 1.51E-05 | 3.49E-03 | -0.645 |
| glutamate | 2.065 | 9.73E-03 | 5.51E-02 | 0.167 |
| methionine | 2.648 | 7.29E-04 | 1.32E-02 | -0.253 |
| aspartate | 2.795 | 1.03E-03 | 1.54E-02 | -0.515 |
| glucose | 5.242 | 2.55E-04 | 8.03E-03 | -0.207 |
| tyrosine | 6.903 | 3.09E-03 | 2.63E-02 | -0.150 |
| histidine | 7.117 | 5.19E-04 | 1.13E-02 | -0.086 |
| Phenylalanine | 7.342 | 1.14E-03 | 1.59E-02 | -0.150 |
| tryptophan | 7.749 | 2.14E-02 | 9.81E-02 | -0.213 |
| formate | 8.461 | 7.27E-04 | 1.32E-02 | -0.449 |

1. FC: fold change

**Supplementary Figure 24.** a) The aliphatic region of ^1^HNMR spectra from SW48 *KRAS* ^+/+,^ *KRAS* ^Q61H/+^, *KRAS* ^A146T/+^ intracellular extract. Numbers indicate signals corresponding to individual metabolites. 1. 1.isoleucine; 2. leucine; 3.valine; 4. lactate; 5. alanine; 6. acetate; 7.glutamate; 8.glutamine; 9. glutathione; 10. *N*-acetylglutamate; 11.succinate; 12. aspartate; 13.creatine; 14. choline; 15. phosphorylcholine; 16. glycerophosphocholine; 17. betaine; 18.taurine; 19. myo-inositol; 20.glycine; 22.glucose

**Supplementary Figure 24.** b) The aromatic region of ^1^H NMR spectra from SW48 *KRAS* ^+/+,^ *KRAS* ^Q61H/+^, *KRAS* ^A146T/+^ extract. Numbers indicate signals corresponding to individual metabolites. 22. glucose; 23. UDP-*N*-acetylglucosamine; 24. UDP-*N*-acetylgalactosamine; 25. NAD; 26. AMP; 27. tyrosine; 28. phenylalanine; 29. ATP

**Supplementary Figure 25.** a) PCA score plot of the 600MHz ^1^H NMR spectra from extracts of SW48 cell lines with *KRAS* mutations in codons 16 and 146 and their wild type counterpart, b) superimposed NMR spectra of cell lines with the same colour coding as in the PCA plot part a); c) ‘the corresponding heat map display of the 600 MHz ^1^H NMR spectra from 0.8 to 10.0 ppm of SW48 cell lines. Red and blue elements in the spectra indicate NMR signals that are more intense, or less intense, respectively, than the median signal intensity for all the samples. **Supplementary Figure 26.** a). MMC score plot of the 600MHz ^1^NMR spectra from extracts of SW48 cells with *KRAS* mutations in codons 61 and 146 and their wild type counterpart, b) superimposed NMR spectra of SW48 cells, with the same colour coding as in the MMC plot; c) the corresponding ‘heat map display of the 600 MHz ^1^H NMR spectra of Red and blue elements in the spectra indicate NMR signals that are more intense, or less intense, respectively, than the median signal intensity for all the samples.

**Supplementary Figure 27.** Leave one out cross validation of the 600 MHz ^1^H NMR spectra from extracts of SW48 cell lines with *KRAS* mutations in codons 61 and 146 using the quadratic as a classifier; The upper left and upper right diagnostic plots indicate the changes in the percentage of correct and the variation related to discrimination as a function of the number of discriminating components respectively. The red color-bar highlights the optimum number of discriminating components. The diagonal of each confusion matrix shows the percentage of samples that are correctly predicted in each class by colouring each box in confusion matrices, black to white with a descending predictivity. The off-diagonal boxes represent the percentage of samples in one class being incorrectly predicted into other classes.

 **Supplementary Figure 28.** At bottom a ‘heat map display of the 600 MHz ^1^H NMR spectra of *KRAS* ^A146T/+^ (top 5 strips) vs the corresponding spectra of *KRAS*^+/+^ (bottom 4 strips). Red and blue elements in the spectra indicate NMR signals that are more intense, or less intense, respectively, than the median signal intensity for the whole samples. At top, the corresponding ANOVA plot, showing positive peaks for those metabolite signals that are more intense in *KRAS* ^A146T/+^, and negative peaks for those metabolite signals that are less intense. The signals are colour coded by the p value adjusted for an FDR of 0.1.

 **Supplementary Figure 29.** At bottom a ‘heat map display of the 600 MHz ^1^H NMR spectra of *KRAS* ^Q61H/+^ (top 5 strips) vs the corresponding spectra of *KRAS*^+/+^ (bottom 4 strips). Red and blue elements in the spectra indicate NMR signals that are more intense, or less intense, respectively, than the median signal intensity for the whole samples. At top, the corresponding ANOVA plot, showing positive peaks for those metabolite signals that are more intense in *KRAS* ^Q61H/+^, and negative peaks for those metabolite signals that are less intense. The signals are colour coded by the p value adjusted for an FDR of 0.1.

**Supplementary Figure 30.** At bottom a ‘heat map display of the 600 MHz ^1^H NMR spectra of *KRAS* ^A146T/+^ (top 5 strips) vs the corresponding spectra of *KRAS*^Q61H/+^ (bottom 5 strips). Red and blue elements in the spectra indicate NMR signals that are more intense, or less intense, respectively, than the median signal intensity for the whole samples. At top, the corresponding ANOVA plot, showing positive peaks for those metabolite signals that are more intense in *KRAS* ^A146T/+^, and negative peaks for those metabolite signals that are less intense. The signals are colour coded by the p value adjusted for an FDR of 0.1.

**Supplementary Table 17.** Summary of the most significant metabolites differentiating between SW48 cells with *KRAS* mutations in codons 61 and 146 and their wild type counterpart

| Metabolite | Chemical shift | *KRAS A146T/+ vs KRAS+/+* | *KRAS Q61H/+* *vs* *KRAS*+/+ | *KRAS A146T/+ vs KRAS Q61H/+* |
| --- | --- | --- | --- | --- |
| isoleucine | 0.943 (t), 1.01 (d), 3.675 (d) | ⭣ | ⭣ | - |
| leucine | 0.961 (d), 0.972 (d), 1.691 (m), 1.720 (m), 1.748 (m) , 3.737 (dd) | ⭣ | ⭣ | - |
| valine | 0.996 (d), 1.046 (d), 3.615 (d) | ⭣ | ⭣ | - |
| lactate | 1.331 (d), 4.113 (q) | - | ⭣ | ⭡ |
| threonine | 1.335 (d), 3.591 (d) | - | ⭣ | ⭡ |
| alanine | 1.48 (d), 3.787 (q) | ⭣ | ⭣ | - |
| acetate | 1.919 (s) | ⭣ | - | - |
| glutamate | 2.059 (m), 2.140 (m), 2.355 (m), 3.761 (dd) | - | - | - |
| UDP-*N*-acetylglucosamine | 2.084 (s), 4.295 (m), 4.36 (m), 5.523 (dd), 5.97 (d), 5.991 (d), 7.96 (d) | ⭣ | ⭣ | - |
| glutamine | 2.145 (m), 2.46 (m), 3.783 (t) | ⭣ | ⭣ | - |
| glutathione | 2.171 (m), 2.560 (m),  2.935 (dd), 2.980 (dd),  3.782 (m), 4.572 (dd) | ⭣ | ⭣ | - |
| succinate | 2.406 (s) | - | ⭣ | - |
| aspartate | 2.684 (dd), 2.816 (dd), 3.902 (dd) | ⭣ | - | ⭣ |
| creatine phosphate | 3.045 (s), 3.95 (s) | ⭡ | ⭣ | ⭡ |
| choline | 3.205 (s) | ⭣ | ⭣ | ⭣ |
| phosphocholine | 3.224 (s), 3.597 (s), 4.17 (m) | - | ⭣ | ⭡ |
| glycerophosphocholine | 3.234 (s), 4.33 (m) | ⭣ | ⭣ | - |
| taurine | 3.27 (t), 3.425 (t)   \|  \|  \| \| --- \| --- \| | ⭡ | - | ⭡ |
| myo-inositol | 3.284 (t), 3.54 (dd), 3.62 (dd), 4.067 (t) | ⭡ | - | ⭡ |
| glucose | 4.65(d),5.2(d) | ⭣ | ⭣ | - |
| GTP | 5.945 (d), 8.14 (s) | ⭡ | - | ⭡ |
| UMP | 5.97 (d), 8.11 (d) | ⭣ | ⭣ | - |
| NAD | 6.044 (d), 6.09 (d), 8.429 (s), 9.15, 9.34 | - | ⭣ | ⭡ |
| fumarate | 6.521 (s) | ⭡ | - | - |
| tyrosine | 6.91 (m), 7.20 (m) | ⭣ | ⭣ | - |
| phenylalanine | 7.34 (d), 7.381 (m), 7.43 (m) | ⭣ | ⭣ | ⭡ |
| AMP | 8.273 (s), 8.614 (s) | - | ⭣ | - |
| ATP | 8.275 (s), 8.538 (s) | ⭡ | ⭣ | ⭡ |

a b
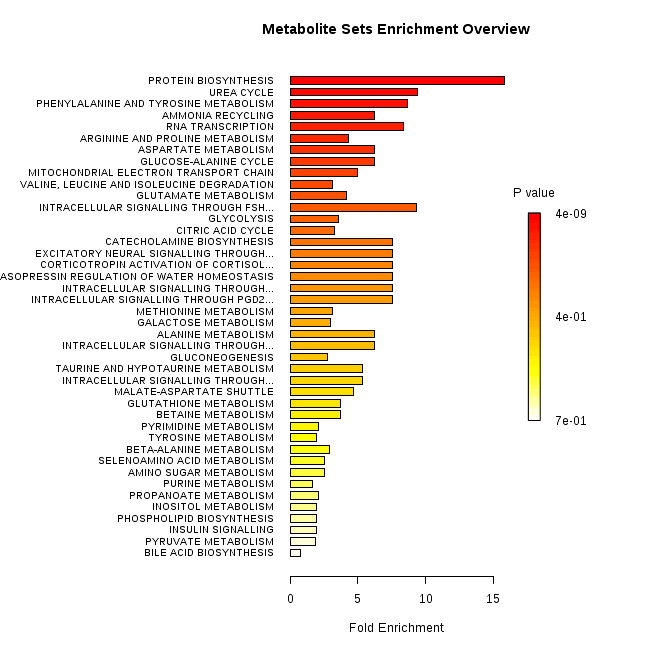

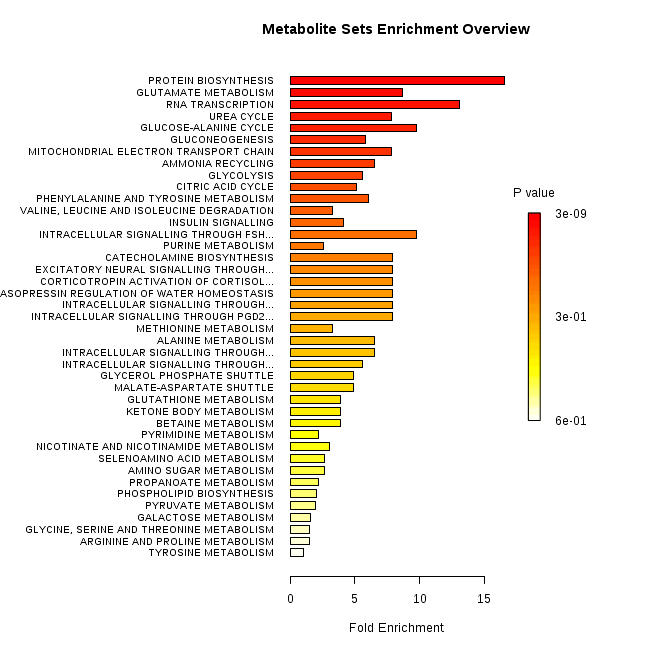


**Supplementary Figure 31.** Metabolic pathway analyses related to the metabolites that were discriminating between a) *KRAS ^A146T/+^*and *KRAS^+/+^* ; b) *KRAS ^Q61H/+^*and *KRAS^+/+^*. The horizontal bars summarize the main metabolite sets identified in this analysis; the bars are coloured based on their p-values and the length is based on the fold enrichment

| Metabolite | ppm | p-values | q-values | log2 FC^1^ |
| --- | --- | --- | --- | --- |
| leucine | 0.966 | 1.45E-03 | 1.06E-02 | -0.341 |
| isoleucine | 1.009 | 1.23E-02 | 5.05E-02 | -0.250 |
| valine | 1.041 | 6.87E-03 | 3.18E-02 | -0.253 |
| alanine | 1.478 | 6.17E-05 | 1.60E-03 | -0.883 |
| acetate | 1.920 | 1.79E-03 | 1.23E-02 | -0.425 |
| glutamine | 2.440 | 6.76E-06 | 3.73E-04 | -1.260 |
| glutathione | 2.544 | 1.31E-02 | 5.28E-02 | -0.189 |
| aspartate | 2.704 | 2.51E-04 | 3.90E-03 | -0.382 |
| creatine phosphate | 3.045 | 1.40E-02 | 5.59E-02 | 0.539 |
| choline | 3.207 | 2.23E-06 | 2.30E-04 | -0.531 |
| glycerophosphocholine | 3.233 | 2.18E-07 | 7.64E-05 | -1.368 |
| taurine | 3.428 | 4.66E-04 | 5.62E-03 | 0.675 |
| myo-inositol | 4.067 | 6.72E-04 | 6.87E-03 | 0.439 |
| glucose | 5.235 | 9.50E-05 | 2.11E-03 | -0.324 |
| UDP-*N*-acetylglucosamine | 5.513 | 3.24E-06 | 2.65E-04 | -0.214 |
| fumarate | 6.521 | 1.43E-02 | 5.68E-02 | 0.152 |
| tyrosine | 6.914 | 3.47E-03 | 1.89E-02 | -0.087 |
| phenylalanine | 7.329 | 1.21E-03 | 9.58E-03 | -0.155 |
| UMP | 8.105 | 3.74E-03 | 2.00E-02 | -0.193 |
| GTP | 8.139 | 2.29E-03 | 1.44E-02 | 0.107 |
| ATP | 8.537 | 2.06E-03 | 1.34E-02 | 0.242 |

**Supplementary Table 18.** Intracellular metabolites discriminating between *KRAS^A146T /+^* and *KRAS^+/+^*

1. FC: fold change

**Supplementary Table 19.** Intracellular metabolites discriminating between *KRAS ^Q61H/+^* and *KRAS^+/+^*

| Metabolite | ppm | p-values | q-values | log 2 FC^1^ |
| --- | --- | --- | --- | --- |
| leucine | 0.966 | 1.00E-06 | 1.95E-04 | -0.582 |
| isoleucine | 1.009 | 3.66E-05 | 1.37E-03 | -0.441 |
| valine | 1.041 | 6.05E-06 | 4.74E-04 | -0.464 |
| lactate | 1.325 | 6.01E-05 | 1.83E-03 | -1.148 |
| threonine | 1.329 | 5.74E-06 | 4.62E-04 | -0.542 |
| alanine | 1.479 | 4.53E-05 | 1.56E-03 | -0.939 |
| succinate | 2.406 | 7.29E-06 | 5.25E-04 | -0.875 |
| glutamine | 2.44 | 1.03E-07 | 9.11E-05 | -1.175 |
| glutathione | 2.544 | 1.67E-04 | 3.72E-03 | -0.186 |
| creatine phosphate | 3.045 | 3.46E-05 | 1.34E-03 | -0.713 |
| choline | 3.208 | 1.97E-02 | 8.16E-02 | -0.185 |
| phosphocholine | 3.225 | 3.00E-03 | 2.41E-02 | -0.229 |
| glycerophosphocholine | 3.234 | 1.81E-07 | 9.76E-05 | -1.360 |
| glucose | 5.235 | 2.35E-04 | 4.72E-03 | -0.291 |
| UDP-*N*-acetylglucosamine | 5.513 | 2.87E-05 | 1.19E-03 | -0.223 |
| NAD | 6.050 | 5.69E-03 | 3.53E-02 | -0.103 |
| tyrosine | 6.914 | 1.26E-05 | 7.01E-04 | -0.196 |
| phenylalanine | 7.329 | 2.94E-04 | 5.46E-03 | -0.248 |
| UMP | 8.105 | 4.04E-04 | 6.81E-03 | -0.219 |
| ATP | 8.537 | 1.28E-02 | 5.97E-02 | -0.141 |
| AMP | 8.605 | 4.52E-03 | 3.08E-02 | -0.301 |

1. FC: fold change

**Supplementary Information References**

Arena, S., Pisacane, A., Mazzone, M., Comoglio, P. M. & Bardelli, A. (2007) Genetic targeting of the kinase activity of the Met receptor in cancer cells. *Proceedings of the National Academy of Sciences of the United States of America*, 104(27), 11412-11417.

Benjamini, Y. (2010) Discovering the false discovery rate. *Journal of the Royal Statistical Society Series B-Statistical Methodology*, 72, 405-416.

Dieterle, F., Ross, A., Schlotterbeck, G. & Senn, H. (2006) Probabilistic quotient normalization as robust method to account for dilution of complex biological mixtures. Application in 1H NMR metabonomics. *Anal Chem*, 78(13), 4281-90.

Han, B., Kang, H. M. & Eskin, E. (2009) Rapid and Accurate Multiple Testing Correction and Power Estimation for Millions of Correlated Markers. *PLoS Genetics*, 5(4), e1000456.

Varshavi, D., Scott, F. H., Varshavi, D., Veeravalli, S., Phillips, I. R., Veselkov, K., Strittmatter, N., Takats, Z., Shephard, E. A. & Everett, J. R. (2018) Metabolic Biomarkers of Ageing in C57BL/6J Wild-Type and Flavin-Containing Monooxygenase 5 (FMO5)-Knockout Mice. *Frontiers in Molecular Biosciences*, 5, 28.

Vartanian, S., Bentley, C., Brauer, M. J., Li, L., Shirasawa, S., Sasazuki, T., Kim, J.-S., Haverty, P., Stawiski, E., Modrusan, Z., Waldman, T. & Stokoe, D. (2013) Identification of Mutant K-Ras-dependent Phenotypes Using a Panel of Isogenic Cell Lines. *Journal of Biological Chemistry*, 288(4), 2403-2413.

Veselkov, K. A., Mirnezami, R., Strittmatter, N., Goldin, R. D., Kinross, J., Speller, A. V. M., Abramov, T., Jones, E. A., Darzi, A., Holmes, E., Nicholson, J. K. & Takats, Z. (2014) Chemo-informatic strategy for imaging mass spectrometry-based hyperspectral profiling of lipid signatures in colorectal cancer. *Proceedings of the National Academy of Sciences of the United States of America*, 111(3), 1216-1221.

Veselkov, K. A., Vingara, L. K., Masson, P., Robinette, S. L., Want, E., Li, J. V., Barton, R. H., Boursier-Neyret, C., Walther, B., Ebbels, T. M., Pelczer, I., Holmes, E., Lindon, J. C. & Nicholson, J. K. (2011) Optimized preprocessing of ultra-performance liquid chromatography/mass spectrometry urinary metabolic profiles for improved information recovery. *Anal Chem*, 83(15), 5864-72.
